# Global phylodynamic analysis of avian paramyxovirus-1 provides evidence of inter-host transmission and intercontinental spatial diffusion

**DOI:** 10.1186/s12862-019-1431-2

**Published:** 2019-05-24

**Authors:** Joseph T. Hicks, Kiril M. Dimitrov, Claudio L. Afonso, Andrew M. Ramey, Justin Bahl

**Affiliations:** 1 Department of Infectious Diseases, College of Veterinary Medicine, University of Georgia, 501 D. W. Brooks Drive, Athens, GA 30602 USA; 2 Exotic and Emerging Avian Viral Disease Research Unit, Southeast Poultry Research Laboratory, US National Poultry Research Center, ARS, USDA, Athens, GA USA; 3 US Geological Survey, Alaska Science Center, Anchorage, AK USA; 4 Program in Emerging Infectious Diseases, Duke-National University of Singapore Graduate Medical School, 8 College Road, Singapore, 169857 Singapore

**Keywords:** Phylogeography, Newcastle disease, Viral disease ecology, Viral migration

## Abstract

**Background:**

Avian avulavirus (commonly known as avian paramyxovirus-1 or APMV-1) can cause disease of varying severity in both domestic and wild birds. Understanding how viruses move among hosts and geography would be useful for informing prevention and control efforts. A Bayesian statistical framework was employed to estimate the evolutionary history of 1602 complete fusion gene APMV-1 sequences collected from 1970 to 2016 in order to infer viral transmission between avian host orders and diffusion among geographic regions. Ancestral states were estimated with a non-reversible continuous-time Markov chain model, allowing transition rates between discrete states to be calculated. The evolutionary analyses were stratified by APMV-1 classes I (*n* = 198) and II (*n* = 1404), and only those sequences collected between 2006 and 2016 were allowed to contribute host and location information to the viral migration networks.

**Results:**

While the current data was unable to assess impact of host domestication status on APMV-1 diffusion, these analyses supported the sharing of APMV-1 among divergent host taxa. The highest supported transition rate for both classes existed from domestic chickens to Anseriformes (class I:6.18 transitions/year, 95% highest posterior density (HPD) 0.31–20.02, Bayes factor (BF) = 367.2; class II:2.88 transitions/year, 95%HPD 1.9–4.06, BF = 34,582.9). Further, among class II viruses, domestic chickens also acted as a source for Columbiformes (BF = 34,582.9), other Galliformes (BF = 34,582.9), and Psittaciformes (BF = 34,582.9). Columbiformes was also a highly supported source to Anseriformes (BF = 322.0) and domestic chickens (BF = 402.6). Additionally, our results provide support for the diffusion of viruses among continents and regions, but no interhemispheric viral exchange between 2006 and 2016. Among class II viruses, the highest transition rates were estimated from South Asia to the Middle East (1.21 transitions/year; 95%HPD 0.36–2.45; BF = 67,107.8), from Europe to East Asia (1.17 transitions/year; 95%HPD 0.12–2.61; BF = 436.2) and from Europe to Africa (1.06 transitions/year, 95%HPD 0.07–2.51; BF = 169.3).

**Conclusions:**

While migration appears to occur infrequently, geographic movement may be important in determining viral diversification and population structure. In contrast, inter-order transmission of APMV-1 may occur readily, but most events are transient with few lineages persisting in novel hosts.

**Electronic supplementary material:**

The online version of this article (10.1186/s12862-019-1431-2) contains supplementary material, which is available to authorized users.

## Background

Avian avularvirus 1 whose isolates are known as avian paramyxoviruses 1 (APMV-1, used hereafter) [1], is a diverse viral agent whose virulent forms cause Newcastle disease (ND), a globally distributed avian disease that affects both wild and domestic birds [2, 3]. First described in 1926, APMV-1 are of varying virulence in avian hosts from the asymptomatic “lentogenic” strains to the highly virulent “velogenic” strains that can cause gastrointestinal and neurological disease. During the containment efforts of the Newcastle disease outbreak within the United States in 2002 to 2003, 3.16 million birds were depopulated, at an estimated cost of US$281 million in total direct and indirect losses [4]. Economic impacts of ND in poultry occur not only from mortality and depopulation of stock, but also from preventative measures and restriction of poultry trade during and immediately following outbreaks [5–8].

Phylogenetically, APMV-1 is divided into two groups, namely class I and class II. Class I isolates are classified as a single genotype with 4 sub-genotypes while the much more diverse class II isolates are divided into 18 genotypes, many of which are further differentiated into sub-genotypes [3]. Some genotypes appear limited in host and geographic distribution, and others such as class II genotypes I, V, VI and VII have been isolated in many countries and avian species, suggesting viral dispersion across geographic areas and among diverse hosts [3, 9]. While APMV-1 has been reported to infect birds from as many as 27 taxonomic orders through either natural or experimental means, it is believed that many more (and perhaps all) bird species are susceptible to infection [6]. Transmission between host species appears to be an important mechanism contributing to the maintenance, propagation, and spread of APMV-1, potentially influenced by the bi-directionality of viral exchange between domestic and wild bird populations. For example, previous research efforts support the exchange of virus between wild and domestic flocks, including the diffusion of live vaccine strains intended for domestic poultry into wild birds [10, 11]. The majority of APMV-1 maintained in wild birds are predicted to be avirulent in gallinaceous poultry [2, 12], but experimental evidence has shown that multiple passages of avirulent wild APMV-1 in chickens can produce virulent APMV-1 strains [13]. APMV-1 of low virulence are capable of evolving naturally into a virulent phenotype; however, this has been documented only occasionally [14, 15]. This suggests that though the initial transmission of wild bird virus into a domestic population may not have significant epidemiological consequences, the repeated transference of virulent viruses between domestic and wild birds likely affects global APMV-1 dynamics. Even though other investigations have found evidence for APMV-1 migration between hosts and geographic regions [9, 12, 16–19], a globally-distributed analysis of APMV-1 viral migration has not yet been performed. Presence of virulent APMV-1 in poultry is reportable to the World Organisation for Animal Health (OIE) and may result in trade restrictions [20]; therefore, understanding the patterns of viral migration between world regions and host types would be useful for efforts directed towards preventing and limiting the spread of ND among wild and domestic birds.

In this study we aim to quantify viral transmission of APMV-1 across avian hosts and geographic regions with the goal of elucidating the global transmission network of these economically important viruses. By identifying these pathways of viral movement, our analysis may reveal critical points that can be targets of control, prevention, and surveillance efforts. Building on evidence that the evolution of APMV-1 is structured by both geography and host order [12], a comprehensive phylodynamic analysis allows for the reconstruction of host and location history and enables the estimation of viral migration rates. Although limited reporting of the ecological origin (i.e., wild vs. domestic origin) of avian hosts, especially waterfowl, prevents the direct observation of the effect of domestication status on viral diffusion, rates and counts between avian orders, world geographic regions and into, out of and within the United States provide evidence to assess the relative importance of interspecies transmission and migration in shaping both the long-term and short-term genetic structure of the viral population.

## Results

### Host and geographic characteristics

A total of 1602 complete fusion gene coding sequences of APMV-1 collected between 1970 and 2016 were included in the final analysis (accession numbers available in public GitHub repository, https://github.com/jt-hicks/Hicks-et-al_2018_APMV-1). The 1404 class II sequences represented the entire time period of inclusion with the majority of these viruses collected between 1997 and 2016. All 198 class I sequences were collected after 1997. While this dataset is globally distributed and represents the most comprehensive APMV-1 data available, due to a lack of systematic APMV-1 surveillance and sequencing, many countries with recent Newcastle disease outbreaks are not represented within this analysis (Additional file 2: Figure S1-A). The represented nations do encompass the majority of areas high in domestic chicken and duck density (Additional file 2: Figure S1-B&C) [21]. Furthermore, given the temporal distribution of sequences analyzed, those originating from samples collected from 1970 to 2016 were included in the phylogenetic estimation, but only sequences from samples collected after 2005 were included in the host and location reconstruction in order to focus the analysis on recent years of APMV-1 activity. Sequences collected before 2006 (class I = 24 sequences; class II = 337 sequences) are henceforth referred to as “older sequences.”

Sequences were categorized by the sampled host taxonomic order. Unfortunately, domestication status and ecological origin of the reported hosts were often unclear or unknown preventing the inclusion of this important data into the described models. Class I viruses collected between 2006 and 2016 originated from Anseriformes (122), Charadriiformes (12), domestic chickens (31), Pelecaniformes (2), other Galliformes (1), Columbiformes (2), and Accipitriformes (1) hosts. Along with three class I sequences without a recorded host, the sequences collected from the latter four host groups were categorized into an ambiguous group (i.e., “other avian taxa” henceforth) given small sample sizes. The majority of class II viruses collected between 2006 and 2016 originated from Anseriformes (171), domestic chickens (569), Columbiformes (220), other Galliformes (34), Psittaciformes (11), and Suliformes (19) hosts. Similar to class I, 43 class II sequences from rare or unrecorded host groups were collected into the other avian taxa category. Specifically, this group was made up of Accipitriformes (7), Bucerotiformes (1), Charadriiformes (5), Coraciiformes (3), Gruiformes (3), mammal (1), Passeriformes (4), Pelecaniformes (4), Strigiformes (1), Struthioniformes (2), and vague/unrecorded host (12) (Additional file 1: Table S1).

Class I viruses collected between 2006 and 2016 originated from wild and domestic birds sampled in East Asia (92), Europe (3), Central Asia (1) and North America (77). Class II viruses were isolated from samples collected in Africa (218), Central America (21), Central Asia (10), East Asia (416), Europe (45), the Middle East (75), North America (142), South America (18), South Asia (109), and Southeast Asia (9) (Additional file 1: Table S2). A single class I and 4 class II sequences originated from an unspecified location within Russia and so were treated ambiguously among East Asia, Europe and Central Asia.

The 77 class I viruses isolated from samples collected in the United States between 2006 and 2016 consisted of 31 Alaska sequences, 20 Midwest sequences, 17 Northeast sequences, 8 South sequences, and 2 West sequences. Of the 142 class II viruses isolated in the United States between 2006 and 2016, 8 originated from Alaska, 35 from the Midwest, 61 from the Northeast, 8 from the Plains, 22 from the South, 1 from the West and 7 without specific state location identified (Additional file 1: Table S3).

### Phylogenetic analysis

Upon estimation of the phylogenetic history, class I viruses were organized into three main lineages which correspond to previously identified sub-genotypes. Sub-genotypes 1a and 1b share a common ancestor, though they represent distinct clades from each other (posterior probability (pp) = 1.0 for both clades). Sub-genotypes 1c and 1d, combined, represent a distinct lineage (pp = 0.996) that diverged from genotypes 1a and 1b about 51 years ago (95% HPD 33.6–73.6) (Additional file 1: Table S4).

The class II APMV-1 represent a much more diverse sample with a time to the most recent common ancestor (TMRCA) of 136.3 years ago (95% highest posterior density (HPD) 112.3–161.7) (Additional file 1: Table S4). TMRCA of class II genotypes ranged from 19.0 years ago to 85.8 years ago (genotype XI 95% HPD 13.1–28.4; genotype IV 95% HPD 64.9–115.1). When compared to previous genotype identification, all genotypes represented in this dataset produced monophyletic, well-supported lineages except for genotype IV which did not exist in a single monophyletic clade. Rather, genotype IV taxa were estimated (pp = 1.0) to belong to a lineage with genotype XI, itself a monophyletic subgroup (pp = 1.0). Of the class II genotypes in this analysis, only genotypes III, IV, VIII, and IX were not present in the data set after 2000, suggesting extinction of the viruses of these lineages.

### Host dynamics

**Fig. 1:**
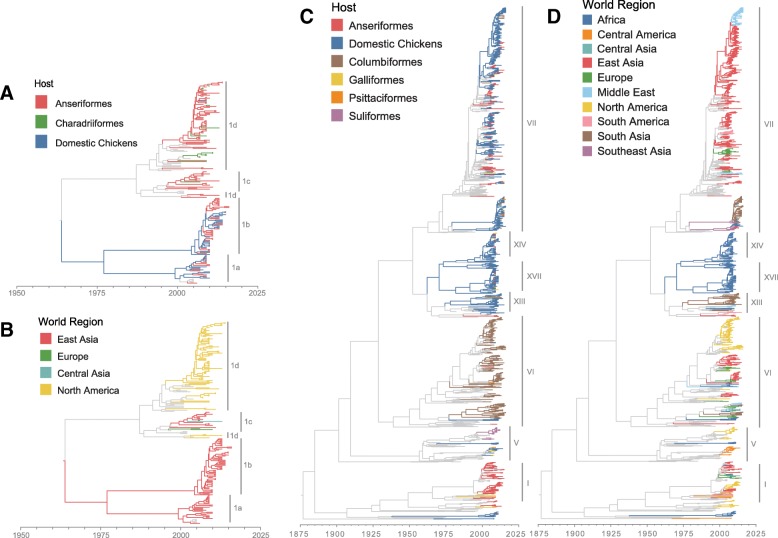
Maximum clade credibility phylogenetic trees with ancestral state reconstruction of host order (**a** – class I; **c** – class II) and world geographic region (**b** – class I; **d** – class II) ecologic models. Important sub-genotypes (class I) and genotypes (class II) are indicated with vertical gray bars

**Fig. 2:**
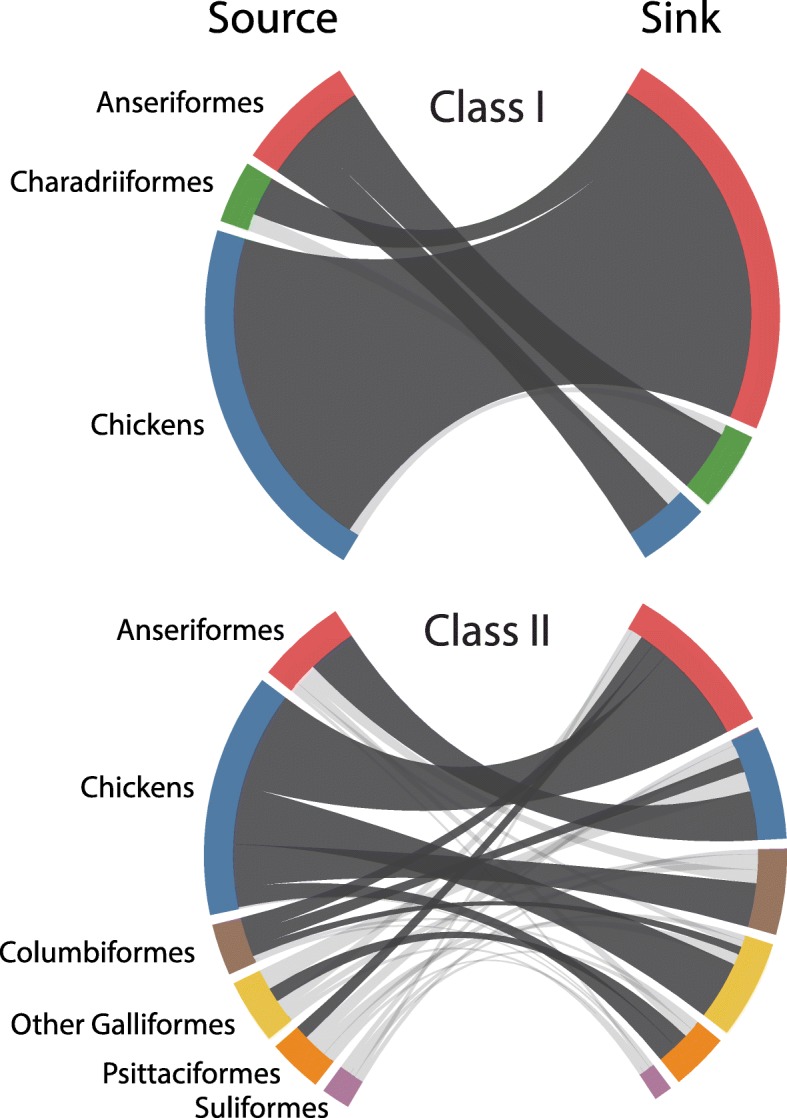
Class I and class II chord diagrams representing the fully resolved transtition matrix between host orders. Chord width between source and sink host state is proportional to the median transition rate per year. Dark gray chords are statistically supported (BF > 3.0). Colors correspond to host order: Anseriformes – red, Charadriiformes – green, domestic chickens – blue, Columbiformes – brown, other Galliformes – yellow, Psittaciformes – orange, Suliformes – purple

Ancestral reconstruction of class I viral host states provides support for differences in host structuring by sub-genotype (Fig. 1a). Sub-genotypes 1c and 1d were primarily collected from Anseriformes hosts. While the host of the most recent common ancestor of these sub-genotypes could not be estimated, more recent lineages provide evidence for several independent transmission events from Anseriformes into Charadriiformes and domestic chickens. The highest supported rate of transmission for this model is that from Anseriformes to Charadriiformes (median = 1.16 transitions per year; 95% HPD 0.30–2.27; Bayes factor (BF) = 11,050.9) (Fig. 2i, Additional file 1: Table S5). The reverse of this rate (from Charadriiformes to Anseriformes) has much lower support (0.74 transitions per year; 95% HPD 0.0–2.23; BF = 13.0). Class I sub-genotypes 1a and 1b also have a complex host history with a large amount of viral exchange between domestic chickens and Anseriformes. The majority of ancestral nodes within these sub-genotypes are estimated as existing within domestic chickens, yet none rise to the level of statistical support. This uncertainty may indicate ease of transmission between domestic chickens and Anseriformes, which is further supported by high transition rates between the two (chicken to Anseriformes: 6.18 transitions per year, 95% HPD 0.31–10.02, BF = 367.2; Anseriformes to chickens: 1.16 transitions per year, 95% HPD 0.30–2.27, BF = 11,050.9). In contrast, there was no support for transitions between chickens and Charadriiformes in either direction (Fig. 2i, Additional file 2: Figure S2), suggesting that these avian groups do not exchange class I APMV-1.

The viral host history for class II isolates appeared to vary by genotype (Fig. 1c). Generally, the ancestral history of class II viruses is structured by host, with host types clustering together within genotype clades. For instance, genotypes XII, XIII, XIV, XVII and XVIII were predominantly detected in domestic chickens with evidence for transitions to other hosts including Anseriformes, Psittaciformes, Columbiformes and other Galliformes birds. In contrast, genotypes I and VI were mostly detected in non-chicken hosts, such as Anseriformes and Columbiformes, respectively. Taken as a whole, of the 30 possible transition rates between host orders in the class II model, 10 (33%) were statistically significant (BF > 3.0). The greatest rates of change with Bayesian support for the class II viruses were from chickens to Anseriformes (2.88 transitions per year; 95% HPD 1.91–4.06; BF = 34,582.9), Anseriformes to chickens (1.44 transitions per year; 95% HPD 0.52–2.74; BF = 34,582.9), chickens to other Galliformes (1.73 transitions per year; 95% HPD 1.06–2.60; BF = 34,582.9), and chickens to Columbiformes (1.29 transitions per year; 95% HPD 0.68–2.02; BF = 34,582.9) (Fig. 2ii; Additional file 1: Table S6). When transition counts were indirectly observed through time between 2005 and 2016, domestic chickens provided a persistent source of virus for Anseriformes, Columbiformes and other Galliformes birds (Additional file 2: Figure S3). Anseriformes also provided a persistent source of virus for domestic chickens. In contrast, Columbiformes appeared to be an inconsistent source of APMV-1 between 2005 and 2016 for Anseriformes, chickens and other Galliformes birds; that is, Columbiformes acted as a viral source to these groups for only 1–2 year periods in recent years.

### Global migration dynamics

For most sub-genotypes of class I APMV-1, the phylogenetic history is strongly structured on global geography (Fig. 1b). Sub-genotypes 1a and 1b were identified solely in East Asia, specifically China, whereas sub-genotype 1d viruses were collected exclusively in North America. Only viruses of sub-genotype 1c provide evidence of diffusion between global geographic regions with a mixture of viruses collected from Europe, East Asia and Central Asia. When this minimal global diffusion is quantified within the phylodynamic model, only a single rate is statistically supported (East Asia to Europe, 0.47 transitions per year; 95% HPD 0.01–1.37; BF = 18.9) (Additional file 1: Table S7, Additional file 2: Figure S4). This suggests the majority of class I APMV-1global diffusion occurred before 2006 when this analysis begins to indirectly observe host transitions.

**Fig. 3:**
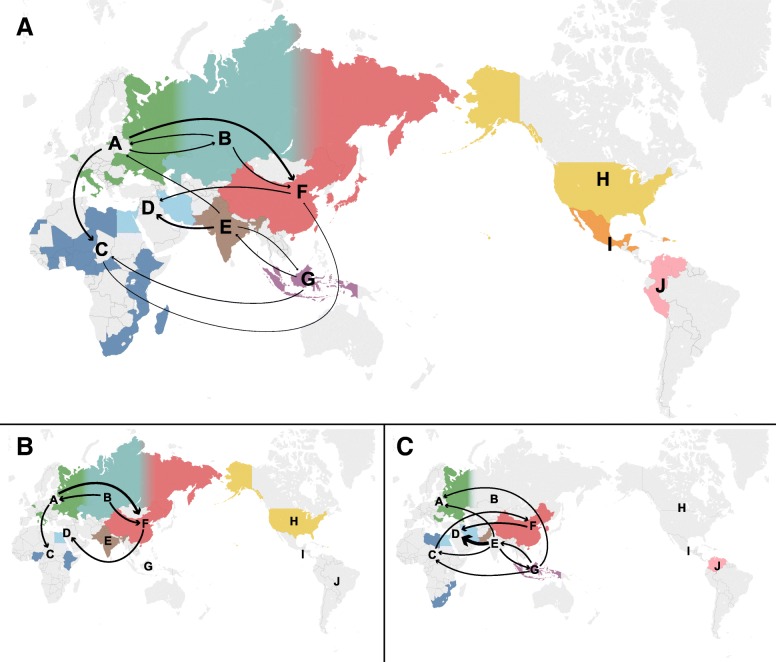
World geographic migration network for all class II viruses (**a**), genotype VI (**b**), and genotype VII (**c**). Arrows represent statistically supported migration rates (BF > 3.0) with width proportional to median migration rate per year. Countries are colored based on sequence representation within the respective model: Europe (A) – green, Central Asia (B) – turquoise, Africa (C) – dark blue, Middle East (D) – light blue, South Asia (E) – brown, East Asia (F) – red, Southeast Asia (G) – grey, Oceania (H) – purple, North America (I) – yellow, Central America and Caribbean (J) – orange, South America (K) – pink. Russian sequences were divided between Europe, Central Asia and East Asia based on actual location of collection. Base map was used with permission from OpenStreetMap (https://www.openstreetmap.org/copyright)

The majority of class II global outbreaks appear to have originated from older, established virus populations and not from viruses introduced from another global region between 2006 and 2016 (Fig. 1d). Despite this, there was also evidence of viral dispersal from endemic regions to new geographic regions resulting in localized epidemics. For example, genotype VII was inferred to migrate into South Asia, East Asia and Africa from Southeast Asia and to the Middle East from East Asia. Most of these genotype VII transition events appear to be successful migrations, resulting in viral lineages specific to the recipient region, rather than a dead-end transition. Of the 90 transition rates assessed in the migration matrix of this size, 11 (12.2%) were statistically supported (BF > 3.0) (Additional file 1: Table S8). The largest rates of migration were measured from South Asia to Middle East (1.21 transitions per year; 95% HPD 0.36–2.45; BF = 67,107.8), Europe to East Asia (1.17 transitions per year, 95% HPD 0.12–2.61; BF = 436.2), and Europe to Africa (1.06 transitions per year, 95% HPD 0.07–2.51; BF = 169.3) (Fig. 3a). Migration patterns from Europe are also reflected in the genotype VI-restricted model, suggesting this genotype drives these transitions in the full class II analysis (Fig. 3b). In contrast, when the global model is restricted to movement among genotype VII viruses, South Asia predominates the diffusion network as a source or sink in five of the eight statistically supported transition rates. The largest of these occurs from South Asia to the Middle East (2.23 transitions per year, 95% HPD 0.57–4.60; BF = 47,475.7). Between 2005 and 2016, dispersal events between regions were relatively rare (< 1.4 events per year) with no region acting as a constant viral source over the observed period (Additional file 2: Figure S5).

### United States migration dynamics

**Fig. 4:**
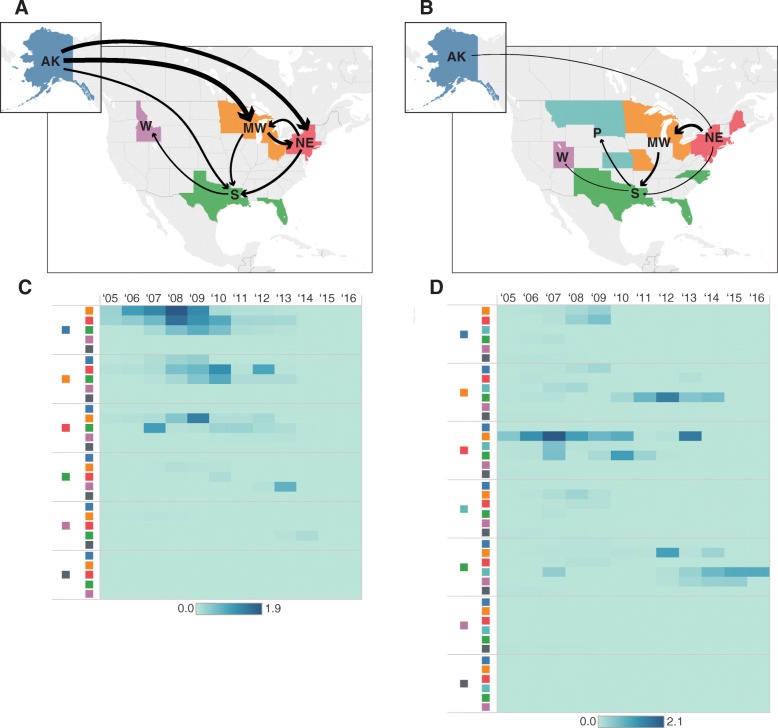
United States region migration rates and count heat map, 2005–2016. Statistically supported migration rates (BF > 3.0) of class I (**a**) and class II (**b**) viruses among regions of the United States are represented by arrows with width proportional to median migration rate per year. In the heat maps of class I (**c**) and class II (**d**) viruses, darkness of each cell is proportional to the absolute number of transitions from the first column into the second column over the most recent 12 years of data. (AK – Alaska, MW – Midwest, NE – Northeast, P – Plains, S – South, W- West, X – Outside United States). Base map was used with permission from OpenStreetMap (https://www.openstreetmap.org/copyright)

No transition rates into or out of the United States were statistically supported during the period of observation, suggesting class I introductions into the country occurred before 2006 (Fig. 4a; Additional file 1: Table S9). Furthermore, our analysis supports Alaska as an important source for class I APMV-1 diversity in the United States. Multiple independent migrations from Alaska into other United States regions were inferred, especially to the Midwest (3.24 transitions per year, 95% HPD 0.95–6.13, BF = 8642.5) and to the Northeast (2.63 transitions per year, 95% HPD 0.58–5.25, BF = 2466.2). Relatively large transition rates were also estimated from the Midwest to the Northeast (1.94 transitions per year; 95% HPD 0.19–4.45; BF = 402.6) and from the Northeast to the South (1.46 transitions per year; 95% HPD 0.09–3.72; BF = 149.5). Since 2005, the peak of class I viral diffusion occurred between 2006 and 2010, coinciding with a period of heavy sampling. During this time, diffusion mainly occurred among Alaska, Northeast and Midwest regions as well as diffusion from these regions into the Southern United States (Fig. 4c).

As with class I APMV-1, the diffusion analysis of sequences collected between 2006 and 2016 provided no evidence of recent migration into or out of the United States for class II APMV-1. Although the largest transition rate within the United States was inferred to be from the Northeast to the Midwest (1.91 transitions per year; 95% HPD 0.77–3.34; BF = 42,728.1), the South also appears to be an important location involved in the recent movement of virus within the United States with 4 supported transition rates involving this region (from the Midwest: 1.35 transitions per year, 95% HPD 0.28–2.69, BF = 62.34; from the Northeast: 0.52 transitions per year, 95% HPD 0.02–1.41, BF = 11.65; to the Plains: 0.85 transitions per year, 95% HPD 0.17–1.84, BF 8541.4; and to the West: 0.33 transitions per year, 95% HPD 0.0–1.03, BF = 6.42) (Additional file 1: Table S10). These rates represent a summary of viral movement of four class II genotypes (I, V, VI, and X) that had been detected within the United States between 2006 and 2016. While some amount of APMV-1 exchange among United States regions was estimated in the ancestral reconstructions of genotype I, V and VI, this finding was in contrast to results of genotype X which was supported to have a geographically structured distribution within the United States. That is, genotype X sequences clustered monophyletically by region. Regional transitions also appeared to shift through time (Fig. 4d). The largest yearly transitions were first inferred as occurring from the Northeast to the Midwest, South and Plains between 2005 and 2010. This was followed by transitions from the Midwest to the South from 2010 to 2014. The South itself then provides transitions to the Plains and the West as well as back to the Midwest between 2012 and 2016.

### Sampling bias

For each model, sensitivity analyses were performed in which sequence traits (host, world region or United States region) were randomly switched throughout the Bayesian analysis. The ancestral root state probabilities (i.e., the probability that the most recent common ancestor, or root, existed in a given host or region) and transition rates of this randomized “tip swap” analysis can then be used to assess the influence of sampling bias in the data. For all three class I models, the ancestral root state probabilities differ considerably between the tip swap analysis and the observed results (Additional file 1: Table S11). Furthermore, the tip swap analysis estimates a different trait as the most probable ancestral root state for each class I model, suggesting the genetic data informs the ancestral reconstruction rather than the sampling scheme. For example, East Asia is estimated as the ancestral root state for class I viruses in the main analysis (pp = 0.72), but the tip swap analysis estimates North America as the ancestral root state (pp = 0.54). In other words, based on the geographic sampling proportions alone, North America would be the most likely origin of all class I APMV-1 diversity with a probability of 0.54. Nevertheless, when the genetic data informs the analysis, the most likely origin of class I APMV-1 diversity was East Asia with a probability of 0.72. For class II APMV-1, though the tip swap analysis and the main analysis predict the same category (older sequences) as the most probable ancestral root state for all three models, the root state probabilities differ among the other categories (Additional file 1: Table S11). For instance, the most recent common ancestor of all class II APMV-1 was inferred to exist in the older sequence category in both the main (pp = 0.84) and tip swap (pp = 0.64) analyses. That being said, the next most probable host for the class II APMV-1 most recent common ancestor was chickens with a probability of 0.22 according to the tip swap analysis, but only 0.03 probability in the main analysis. This suggests that while the ancestral state reconstruction is biased toward older sequences, the genetic data is driving the ancestral reconstruction of the categories of analytical interest (i.e., the unmasked categories in the main analysis).

Examination of the tip swap analysis transition rates provided more detailed evaluation of sampling bias on specific transitions. In an analysis of an unbiased sample, it was expected that the transition rates of a tip swap analysis should approximately equal each other. For all models, most transition rates were similar and estimated to be less than one transition per year, but some rates were estimated to much more than one, indicating the influence of sampling bias. In general, class II host and world region sensitivity analysis transition rates appear less equal to each other than class I rates, though no single host or region is consistently estimated as having a higher tip swap transition rate (Additional file 2: Figures S6 and S7). Except for the transition from Anseriformes to domestic chickens (1.97 transitions per year; 95% HPD 0.22–4.49; Additional file 2: Figure S6), the class I host tip swap analysis transition rates were approximately equal to each other. This large rate may indicate that sampling bias exaggerated the magnitude of the main model’s estimate for class I viral transitions from Anseriformes to domestic chickens. In the class II host model, the highest median rates exist from the older sequence masked category to Anseriformes (2.36 transitions per year; 95% HPD 0.15–4.13) and to chickens (2.19 transitions per year; 95% HPD 0.67–4.90) as well as from chicken to Anseriformes (1.80 transitions per year; 95% HPD 0.46–5.37). In the class I world region model, the highest median transition rates are from North America to older sequences (1.78 transitions per year, 95% HPD 0.04–4.24) and to East Asia (1.24 transitions per year; 95% HPD 0.003–3.41; Additional file 2: Figure S7). In the class II world region model, the highest median rates that do not involve the older masked category exist from East Asia to the Middle East (1.25 transitions per year; 95% HPD 0.25–2.69) and from Europe to Africa (1.11 transitions per year; 95% HPD 0.05–2.74). For the United States region models in both classes, the only rates that are more than 1.5 transitions per year involve the non-United States and older sequences categories (Additional file 2: Figure S8).

## Discussion

This analysis represents the first comprehensive, global phylodynamic study of avian paramyxoviruses 1 to date. As APMV-1 are globally distributed and capable of infecting a broad array of avian hosts, identifying direct epidemiologic links can be difficult. Phylodynamic models estimate the epidemiologic and ecologic characteristics of viral ancestors, thus allowing for the characterization and quantification of viral movement. In the reported analysis, APMV-1 diffusion between avian hosts and geographic regions was quantified. Although the host and world geographic models cannot be directly compared, we found some qualitative differences between the two. The world geographic ancestral reconstruction shows that when APMV-1 is introduced into a location, new regional lineages readily develop. Geographic transitions in general were somewhat rare but were often inferred to result in a successful multi-year outbreak within the recipient region. In contrast, the host ancestral reconstruction was characterized by more frequent and often solitary transitions from an established host into a new recipient host. Host-adapted lineages were inferred to occur, as evidenced by Columbiformes-adapted genotype VI viruses, but were comparatively less common relative to the overall number of observed host transitions. For instance, among class II genotype VII viruses, which were mostly collected from domestic chickens, there are numerous, stuttering transitions into new host orders such as Anseriformes and Columbiformes without the establishment of a persistent lineage within the recipient host. In this sense, these transitions resemble “spill-over” events from a well-established host into a host that is more adaptively challenging to the virus [22]. Ecological barriers that prevent an infected bird from contacting others of its species may also explain unsuccessful continued transition of APMV-1 within a new host group. For example, captive birds such as pet parrots (Psittaciformes) likely become infected with APMV-1 from the live-bird market-like conditions of holding areas prior to export [23]. Exportation and subsequent captivity of these pet birds would limit contact with and viral transmission to individuals of the same species.

Analyses for both class I and class II APMV-1 provide evidence of a viral migration link between domestic chickens and birds of the order Anseriformes, such as ducks, geese, and swans. Anseriform birds exist both in wild habitats and in the domestic poultry industry, potentially acting as a bridge between these two ecologic systems. Though this viral bridging is theoretically possible given the estimated migration networks, viral transfer from strictly wild to strictly domestic populations via waterfowl was not directly observed within the ancestral reconstruction. One explanation is that wild and domestic Anseriformes often represent distinct populations. Evidence of this can be observed in the class I analysis in which the transmission between chickens and Anseriformes appears to occur mostly within domestic East Asian ducks, while transmission between Charadriiformes (an exclusively wild population) and Anseriformes solely involves North American wild ducks. APMV-1 outbreak reports often do not have details on the ecological environment in which the host bird was sampled making it unclear whether a duck is wild, domestic or feral and precluding the formal testing of this hypothesis with the dataset used in this investigation. As evidence exists that domestic and wild ducks often come into contact with one another when domestic ducks are allowed to browse freely [24], the barrier between these two groups may be porous in regions where there is spatiotemporal overlap of infected bird populations, particularly in production systems with low biosecurity. Furthermore, live bird markets may represent a distinct ecological niche that could produce limited cross-species transmission for APMV-1 due to the close proximity of multiple bird species and the constant turnover of naïve hosts.

On the global geographic scale, Europe, East Asia, and South Asia represent important nodes for the global dispersion of APMV-1. While the heavy representation of East Asian viruses (29.6% of all class II sequences) may partially explain this region’s importance within the migration network, the same cannot be argued for South Asian or European viruses, which only represent 3.2 and 7.8%, respectively, of the included class II sequences collected between 2006 and 2016. Live animal movement (i.e., commercial trade and wild bird migration) represent the most likely drivers of global APMV-1 spread although inanimate, vaccine, and non-avian animal contamination may also act as viral migration mechanisms [2, 25–32]. Given the importance of domestic chickens within the host diffusion model, movement of live domestic birds most likely represents an important mechanism for the global dispersion of APMV-1. This is further supported by our findings in which major poultry trade partners are reflected in the global diffusion network. Specifically, Europe, which is a net exporter of poultry to the rest of the world [33], was supported as an important source of class II APMV-1. Moreover, when restricted to genotype VII viruses, which almost exclusively occur within domestic bird populations, viral migration parallels poultry trade routes, such as from South Asia to the Middle East. Poultry trade, however, does not explain all global movement of class II viruses. Rather, as demonstrated by our genotype VI-restricted model and a previously published genotype VI phylodynamic analysis [34], global migration of APMV-1 is also driven by transportation of pigeons, resulting in viral dispersal from Europe to other regions, including Africa and East Asia.

Both the global-scale and North America level analyses provide no evidence for introduction of APMV-1 into the western hemisphere between 2006 and 2016. Rather, our analyses suggest APMV-1 diversity within North America during that period is due to pre-2006 introductions and inter-regional viral movement. Viral diffusion within the United States appears to vary depending on viral class and genotype. A majority of class I APMV-1 were estimated to have been introduced from Alaska into the midwestern, northeastern, and southern United States. In contrast, Alaska may not play an important role in the diffusion of class II APMV-1 within the United States. This difference in regional dynamics may be driven by host type and therefore method of viral transportation. Whereas intercontinental viral dispersal by migrating Anseriform birds via Alaska has been supported by both low-virulence APMV-1 and avian influenza genetic studies [9, 35–37], introduction into the Northeast appears driven by columbiform birds, most likely from racing pigeon trade as opposed to natural migration.

The estimation of APMV-1 diffusion between hosts and geographic regions is also likely influenced by virulence, which varies widely among APMV-1 viruses. Virulence affects both viral epidemiology and evolution [38] and therefore should be considered in phylodynamic analyses. Furthermore, differential distribution of virulent viruses between ecological groups may confound diffusion estimates. Domestic and wild birds differ in the prevalence of virulent APMV-1, exist in differing proportions across the globe and move by different mechanisms. Geography, domestication status, and virulence therefore may interact to produce observed viral diffusion patterns.

A major limitation to any phylodynamic analysis is the dependence on sampling. The tip swap analysis attempted to assess the impact of the viral sampling scheme on the estimated viral diffusion patterns. In this sensitivity analysis, the trait proportions within the sample remain constant, but the traits of individual samples are allowed to randomly change during the Bayesian analysis. As an example, in the host models, the total number of chickens remains constant, but which individual samples are considered as chickens changes throughout the simulation. In an unbiased sample, it is expected that the distribution of ancestral root state probabilities will differ between the main and tip swap analyses. In other words, the estimated probability that the most recent common viral ancestor existed in a particular host or location should be different between the main model and the tip swap analysis. Furthermore, transition rates estimated in the tip swap analysis should be similar to each other. In both classes, the ancestral root state probabilities are markedly different between the two types of analyses, though more so in class I than class II. Both the class II main and tip swap analyses estimate the older sequence category as the most probable state for the most recent common ancestor in each diffusion model. That is, older sequences are estimated as the ultimate origin of class II viruses in all models, even when the analysis ignores the genetic information. This suggests the sample is biased toward the presence of older sequences within the dataset. Evidence of this bias toward older sequences in class II (and to a lesser extent in class I) APMV-1 can be found when comparing the transition rates estimated in the tip swap analysis: the majority of large transition rates (> 1.5 transitions per year) involve the older sequence category. While this may not be ideal, the bias would be toward underestimating transition rates among the hosts and locations remaining in the diffusion model. This is because the older sequence category is masked within the discrete trait diffusion model and so does not contribute to the estimation of rate magnitude or statistical support. For example, within the class II world region model, the ancestral history of genotype VI was predominated by older sequences, preventing the potential detection of viral exchange between such regions as Europe, East Asia, and North America. Nevertheless, this sampling bias toward older sequences may be acceptable as it prevents the estimation of viral transitions further in the past when sampling is sparser and less consistent. The tip swap analysis also provides evidence of sampling bias toward sequences of Anseriformes bird origin in the class I host model. Particularly, the tip swap transition rate from Anseriformes to domestic chickens was inflated above the others. This combined with the lower support for this rate in the main analysis (BF = 10.9) suggests a higher degree of uncertainty for this estimate. In class I APMV-1, viral movement between chickens and waterfowl may be much more unidirectional (i.e., more viral movement from chickens to waterfowl) than the main analysis suggests. Finally, the tip swap transition rates indicate sampling bias toward non-United States sequences in both class I and class II United States region models. Because this would overestimate non-United States regions as a viral source, this bias is unlikely to impact the observed results as no migration into the United States was noted.

Another limitation of our analysis is the reliance on self-submitted data due to a lack of global systematic active surveillance of APMV-1. Viral movement into and out of a host or location can only be measured if viral sequences from that host or location exist. Those countries or birds which have not had surveillance, sampling or viral sequencing performed will not be represented in the migration model. While our sensitivity analysis was able investigate the influence of oversampling, bias from underreporting is difficult to quantify. For example, many countries with recent ND outbreaks are not represented within this analysis (Additional file 2: Figure S1) [39]. Countries in Africa, Europe and the Middle East are particularly underrepresented and so may be more important in the global diffusion of APMV-1 than is currently reported. Despite these limitations, the sequences used in our analyses represent the best available APMV-1 dataset and included information collected from across the globe and representing isolates from numerous bird species. Continued surveillance and sampling, especially among regions with low representation (Central America, Central Asia, South America, South Asia, Southeast Asia, and Oceania) would improve future analyses. Due to sampling limitations identified in the current study, viral migration rates and counts should be interpreted with caution. We recognize that APMV-1 viruses may be dispersed among locations before detection, especially in those time periods of sparse data collection and among poorly sampled locations/hosts. To help mitigate this, sequences collected before 2006 were masked in the discrete trait diffusion models, preventing overestimation of rates from hosts or regions sampled further in the past when surveillance and sequencing were much more unbalanced and sporadic. For instance, our class II dataset’s representation is limited to 7 or less sequences before 1997, mostly collected from domestic chickens and North America, Europe and East Asia.

Despite the potential biases and limitations of the globally available genetic information for APMV-1, molecular epidemiologic analyses that rely on convenience, self-submitted genetic data have been successfully used to investigate viral transmission dynamics and ecology. Such models based on publicly available genetic data have been applied to viral pathogens, including avian influenza virus [33, 40, 41], dengue virus [42], and Ebola virus [43]. In using a similar approach for APMV-1, we strived to provide useful information for future surveillance efforts by identifying hosts and locations with measurable epidemiologic links. For example, the bidirectional viral transitions between domestic chickens and Anseriformes birds for both class I and class II viruses suggest that regardless of APMV-1 phylogenetic classification, poultry sites in close proximity to wild or domestic waterfowl may increase the risk of viral exchange. In addition, based on the inferred exchange of class II viruses between domestic chickens and birds of Columbiformes, Psittaciformes and other Galliformes, the detection of virulent APMV-1 in one of these latter avian orders indicate a need for heightened surveillance and biosecurity efforts among local chicken populations that interact with these birds. Finally, our results have also highlighted the need for additional sampling for and sequencing of APMV-1 isolates in order to improve global viral migration estimates which will improve our ability to understand and potentially mitigate costly viral movement (e.g., via poultry trade).

## Conclusions

This analysis quantifies global patterns of APMV-1 movement, indicating key differences in the role of host and location in viral dispersal. Because migration between regions appears to occur infrequently, geographic location may partially determine APMV-1 diversification and evolution. In contrast, our analysis supports the presence of frequent inter-order host transitions, but most events are transient in nature and unlikely to develop into persistent lineages within novel hosts. By investigating the interplay of viral host and location, patterns in the spread of APMV-1 may help inform global ND prevention measures. Phylodynamic models can be further developed to untangle the ecological influences on global APMV-1 dynamics. Though host and location of virus origin are both important epidemiologic factors, several variables not included in this analysis impact the movement and evolution of viruses. In particular, we recommend that future APMV-1 sequencing and surveillance reports provide the ecological context regarding the sampled host. Helpful data would include host domestication status, not only limited to the dichotomy of domestic vs wild, but also including categories such as peri-domestic, captive/pet, racing (for pigeons), and domestic production system (e.g., commercial industry or backyard poultry). With this added data, knowledge of APMV-1 global and host distribution will be improved, increasing insight into the impact of trade, domestication, migration, and virulence.

## Methods

### Sequencing of viruses

Fifty-four APMV-1 isolates from repositories in Bulgaria (*n* = 2), Egypt (*n* = 27), Hong Kong (*n* = 2), Indonesia (*n* = 2), South Korea (*n* = 1), Mexico (*n* = 4), Pakistan (*n* = 9), USA (*n* = 3), and Vietnam (*n* = 4) were submitted to the Southeast Poultry Research Laboratory of the U.S. Department of Agriculture for further characterization. Viruses were propagated in 9-to-11-day-old specific pathogen-free embryonated chicken eggs [44]. RNA was extracted from infected allantoic fluid using TRIzol LS Reagent (Invitrogen, Carlsbad, CA, USA) following the manufacturer’s instructions. Sanger sequencing with previously described primers [45] or random next-generation sequencing were performed to obtain the complete fusion gene coding sequences as described previously [46, 47].

### Dataset

A curated sequence database of genetic information for the full coding region of the fusion gene for APMV-1 isolates was procured from the U.S. Department of Agriculture Southeast Poultry Research Laboratory. This database was created from all APMV-1 fusion gene sequences publicly available on GenBank, after carefully filtering to remove all vaccine isolates and laboratory spillovers, identical sequences from the same source or outbreak, and sequences showing evidence of recombination events (recombination analysis by RDP4) [48]. Background data for each sequence was collected from the GenBank submission where available, or from corresponding scientific publications. For this analysis, duplicate sequences with identical year, location and host information and all sequences from samples collected prior to 1970 were excluded from the dataset. Sequences collected before 1970 were excluded in order to minimize bias associated with insufficient sampling frequency and uncertainty of early sample quality and handling. Maximum likelihood trees were estimated with RAxML v8.2 [49] for each class using a general time reversible nucleotide substitution model with gamma distribution of rates [50, 51]. The clock signal using these ML trees was investigated with Tempest v1.5 [52]. A class II viral sequence with a clock rate substantially lower than the mean rate (lower 2.5 percentile) was classified as an outlier and removed from subsequent analyses given that such a virus could represent an artifact of sequencing, viral passage mutations or modified vaccine strains.

### Phylogenetic analysis

For classes I and II sequences, BEAST 1.8.4 [53] was used to estimate phylogenetic trees using only nucleotide information (without host or geography data). Both classes used a HKY nucleotide substitution model [54] with gamma distribution of substitution rates, a constant coalescent [55, 56], and a lognormal relaxed molecular clock [57]. A general time reversible (GTR) substitution model has been previously shown to fit APMV-1 genetic data well; however, the HKY model accounts for the most important biological feature of the nucleotide substitution model (i.e., nucleotide transitions and transversions), while maintaining computational tractability when estimating multiple parameters directly from the sequences or from the phylogenetic trees. At least three independent Markov Chain Monte Carlo (MCMC) runs were performed for each class, using a random starting tree for each run. If needed, additional runs were performed to achieve an effective sample size (ESS) of greater than 200 for all critical parameters. Each MCMC ranged from 70 to 100 million states long, sampling every 10,000 states. The time to the most recent common ancestor (TMRCA) was estimated for each represented class II genotype and each class I sub-genotype.

### Phylodynamic models

In order to maximize computational efficiency and to avoid multiple estimations of the nucleotide substitution model, empirical sets of trees were estimated for both class I and class II sequences using the MCMC runs described above. Empirical sets were constructed by combining the last 500 trees of each run. Discrete trait diffusion models were applied to estimate the rate of transition between different hosts and geographic locations. A discrete trait diffusion model allows for the incorporation of ecological data with evolutionary analyses. Homologous to nucleotide substitution models which estimate the rate of nucleotide evolution of genetic sequences, discrete trait models act as a probabilistic substitution model between defined discrete categories (in this analysis, host or location) [58–60]. In this way, these character traits are allowed to “evolve” over time. The discrete trait models used in this analysis use continuous-time Markov models to reconstruct the ancestral history of the discrete trait evolution and estimate the rate of substitution (or transition) between traits while incorporating phylogenetic branch lengths to help inform the probability of transition over time. The Bayesian framework of BEAST provides further benefit by interrogating these models over a changing distribution of phylogenetic trees, as opposed to a fixed tree topology with fixed evolutionary parameters [58]. This bypasses the reliance on a single estimated evolutionary history.

Three separate discrete trait diffusion models were performed on each APMV-1 class. In the host model, viral host was categorized based on avian order, except for the order Galliformes, which was separated into domestic chickens and other galliform birds (such as turkeys, pheasants and quail). Those sequences with missing or vague host information (e.g, a host designation of “avian”) or whose host order was rare within a specific APMV-1 class (i.e., < 1.5%) were treated as an ambiguous state with the potential to exist as any of the other defined categories.

Two separate phylodynamic models based on geography were inferred. The global model categorized the location of collection based on global geographic regions: Africa, Central America, Central Asia, East Asia, Europe, Middle East, North America, Oceania, South America, South Asia, and Southeast Asia. Due to its transcontinental area, Russian sequences were divided between Europe, Central Asia and East Asia, allowing ambiguity between those three regions when an exact location within Russia was not given. The second geographic model focused on the dynamics into and within the United States, categorizing location based on region (Alaska, Midwest, Northeast, Plains, South, West and Non-United States). All viral sequences not collected within the United States were considered in the last category in order to observe diffusion into and out of specific United States regions from the global community at large. United States samples without exact location details were treated as ambiguous between the United States regions present in the model.

The three phylodynamic models (host, world region and United States region) were applied to each class to estimate the ancestral history of host and geographic transitions. We used a non-reversible continuous-time Markov chain model with a strict clock assumption in order to infer directionality of viral transition between host or geographic states [61]. In all phylodynamic models, sequences collected before 2006 were combined into a “masked” category that does not contribute to the diffusion rate estimation. In essence, this allows older sequences to behave as though they were collected from an unknown source. This provides two benefits to the model. First, the sparsity of data from earlier years may disproportionately influence ancestral state reconstructions, biasing the inferred viral source towards those hosts or locations that were heavily sampled before extensive surveillance and sequencing began. Second, this allows diffusion rates to be summarized over the recent past, which is more practical for future APMV-1 surveillance and prevention efforts. The inferred ecological state of a common ancestor was considered statistically supported at a posterior probability (pp) greater than 0.95. Bayesian stochastic search variable selection (BSSVS) was performed to reduce the complexity of the models and to identify significantly non-zero migration rates using a binary indicator (I) [33, 58]. Based on I, Bayes factors (BF) were calculated using SPREAD v1.0 [62]. Rates were considered statistically supported when BF > 3.0. The BF also provides the strength of the evidence based on the following standard levels of support: rates with BF > 3.0 had substantial support, BF > 10.0 had strong support, BF > 30 had very strong support, and BF > 100 had decisive support [63]. The median rates and associated 95% highest posterior density (HPD) were calculated from the non-zero actual rates from the BSSVS analysis using PyMC, a Bayesian statistical python module (https://github.com/pymc-devs/pymc). Because this analysis considers viral movement across many different genotypes that have varying host distributions and epidemiologic behavior, the global migration of class II viruses was further explored by restricting the global discrete diffusion model to genotype VI and genotype VII, separately. Using the same empirical set of phylogenetic trees for the main class II analyses, all sequences from other genotypes were given a single location trait, *X*. The BSSVS analysis was then masked, allowing for the calculation of genotype VI-specific and genotype VII-specific migration rates.

To observe changes in transitions over time, a complete Markov jump history was recorded independent of the BSSVS runs. At least three independent runs of 1 million chain length sampled every 100 states were performed for both BSSVS and the Markov jump history analyses. Due to the increased uncertainty of the ancestral reconstruction farther back in time when sampling was much scarcer, only the Markov jumps between 2005 and 2016 were analyzed. Markov jumps were extracted from the complete jump history using a custom Python script and visualized as heatmaps built using Tableau v10.3 (https://www.tableau.com/.

### Sampling Bias

The influence of sampling bias was assessed by comparing the probability of the estimated ancestral root state from each model with the probability of an ancestral root state obtained when the viral ecological characteristics (host, world region, and United States region) were randomly swapped during the Markov process [59]. When the probabilities are similar between the data-driven and randomized analyses, there is evidence that sampling bias may be driving the observed results. That is, a region may be estimated as a source of the virus only because that region is over-represented in the data. Though sequences collected before 2006 are masked for the BSSVS analysis, the ancestral state reconstruction uses this “older” sequence category in its estimations and so is included as a potential ancestral root state. The transition rates estimated from the tip swap analysis were also used to further assess the influence of sampling bias on individual transition rates. Transition rates between hosts/regions should be roughly equal in an unbiased sample when tip traits are randomly swapped throughout the Markov process. A large transition rate in this scenario may indicate the sampling scheme, as opposed to the genetic sequence data, is driving the observed results.

## Additional files

Additional file 1: **Tables S1 - S11.** (PDF 1191 kb)

Additional file 2: **Figures S1 - S8.** (PDF 8119 kb)

## Abbreviations

95% HPD: 95% highest posterior density
APMV-1: Avian paramyxovirus-1
BF: Bayes factor
BSSVS: Bayesian stochastic search variable selection
ESS: Effective sample size
GTR: General time reversible
MCMC: Markov chain Monte Carlo
ND: Newcastle disease
OIE: World Organisation for Animal Health
pp: Posterior probability
TMRCA: Time to the most recent common ancestor
