# Supplementary material for: Global phylodynamic analysis of avian paramyxovirus-1 provides evidence of inter-host transmission and intercontinental spatial diffusion
Source: BMC Evol Biol. 2019 May 24;19:108. doi: 10.1186/s12862-019-1431-2 (PMC6534909; doi:10.1186/s12862-019-1431-2)
Supplement: Supplementary file 1 — Tables S1 - S11. (PDF 1191 kb) [file 12862_2019_1431_MOESM1_ESM.pdf]

TITLE: Global phylodynamic analysis of avian paramyxovirus-1 provides evidence of inter-host transmission and intercontinental spatial diffusion

AUTHORS: Joseph T. Hicks[1], Kiril M. Dimitrov[2], Claudio L. Afonso[2], Andrew M. Ramey[3], and Justin Bahl[1,4]

[1]University of Georgia College of Veterinary Medicine Department of Infectious Diseases, Athens, Georgia, USA

[2]Exotic and Emerging Avian Viral Disease Research Unit, Southeast Poultry Research Laboratory, US National Poultry Research Center, ARS, USDA, Athens, GA, USA

[3]US Geological Survey, Alaska Science Center, Anchorage, AK, USA

[4]Program in Emerging Infectious Diseases, Duke-National University of Singapore Graduate Medical School, 8 College Road, Singapore 169857, Singapore

For Correspondence Contact: Joseph Hicks: Joseph.Hicks@uga.edu or Justin Bahl: Justin.Bahl@uga.edu

Address: 501 D. W. Brooks Drive, Athens, Georgia, 30602

Please find below descriptions and tables for Additional File 1.

Supplemental Tables

Supplemental Table S1. Categorization of host order by viral class. Number and percentage (per class) of sequences included in the final analysis are given by original order classification. Unspecified orders included “avian,” “wild bird,” “environment,” or missing. (**Page 3**)

Supplemental Table S2. Categorization of world region by viral class. Number and percentage (per class) of sequences included in the final analysis are given by world region. “Eurasia” corresponds to sequences originating in Russia, but without precise location information.(**Page 4**)

Supplemental Table S3. Categorization of United States region by class. Number and percentage (per class) of sequences included in the final analysis are given by US state. “Unspecified USA” corresponds to sequences with no state designation. (**Page 5**)

Supplemental Table S4. Median time to the most recent common ancestor (TMRCA) for each represented APMV-1 genotype. (95% HPD = 95% highest posterior density credibility interval) (**Page 7**)

Supplemental Table S5. Class I median diffusion rates between host orders. The 95% Bayesian credibility interval and Bayes factor (BF) are provided for each rate. Rates are shaded based on the level of BF support. (**Page 8**)

Supplemental Table S6. Class II median diffusion rates between host orders. The 95% Bayesian credibility interval and Bayes factor (BF) are provided for each rate. Rates are shaded based on the level of BF support. (**Page 9**)

Supplemental Table S7. Class I median migration rates between world regions. The 95% Bayesian credibility interval and Bayes factor (BF) are provided for each rate. Rates are shaded based on the level of BF support. (**Page 10**)

Supplemental Table S8. Class II median migration rates between world regions. The 95% Bayesian credibility interval and Bayes factor (BF) are provided for each rate. Rates are shaded based on the level of BF support. (**Page 11**)

Supplemental Table S9. Class I median migration rates into, out of and within the United States. The 95% Bayesian credibility interval and Bayes factor (BF) are provided for each rate. Rates are shaded based on the level of BF support. (**Page 12**)

Supplemental Table S10. Class II median migration rates into, out of and within the United States. The 95% Bayesian credibility interval and Bayes factor (BF) are provided for each rate. Rates are shaded based on the level of BF support. (**Page 13**)

Supplemental Table S11. Root state probabilities of the inferred phylodynamic models versus those of the randomized sensitivity analyses. Bolded rows indicate the trait with the highest root probability within the inferred model. (**Page 14**)

Supplemental Table S1

Class	Code	Order	N	%
I	ANS	Anseriformes	122	61.62
	CHA	Charadriiformes	12	6.06
	CHI	Galliformes	31	15.66
	UN	Accipitriformes	1	0.51
		Columbiformes	2	1.01
		Galliformes	1	0.51
		Pelecaniformes	2	1.01
		Unspecified	3	1.52
	Older sequences		24	12.12
II	ANS	Anseriformes	171	12.18
	CHI	Galliformes	569	40.53
	COL	Columbiformes	220	15.67
	GAL	Galliformes	34	2.42
	PSI	Psittaciformes	11	0.78
	SUL	Suliformes	19	1.35
	UN	Accipitriformes	7	0.50
		Bucerotiformes	1	0.07
		Charadriiformes	5	0.36
		Coraciiformes	3	0.21
		Gruiformes	3	0.21
		Mammal	1	0.07
		Passeriformes	4	0.28
		Pelecaniformes	4	0.28
		Strigiformes	1	0.07
		Struthioniformes	2	0.14
		Unspecified	12	0.85
	Older sequences		337	24.00

Supplemental Table S2

Class	World Region	N	%
I	East Asia	92	46.46
	Europe	3	1.52
	Central Asia	1	0.51
	North America	77	38.89
	Eurasia	1	0.51
	Older Sequences	24	12.12
II	Africa	218	15.53
	Central America	21	1.50
	Central Asia	10	0.71
	East Asia	416	29.63
	Eurasia	4	0.28
	Europe	45	3.21
	Middle East	75	5.34
	North America	142	10.11
	South America	18	1.28
	South Asia	109	7.76
	Southeast Asia	9	0.64
	Older Sequences	337	24.00

Supplemental Table S3

Class	US Region	US State	N	%
I	Alaska	AK	31	15.66
	Midwest	MI	2	1.01
		MN	16	8.08
		OH	1	0.51
		WI	1	0.51
	Northeast	CT	1	0.51
		DE	1	0.51
		NJ	11	5.56
		NY	1	0.51
		PA	2	1.01
	South	FL	1	0.51
		LA	3	1.52
		TX	4	2.02
	West	ID	2	1.01
	Outside USA	N/A	97	48.99
	Older Sequence		24	12.12
II	Alaska	AK	8	0.57
	Midwest	MI	2	0.14
		MN	25	1.78
		MO	1	0.07
		OH	2	0.14
		WI	5	0.36
	Northeast	CT	1	0.07
		DE	1	0.07
		MA	3	0.21
		MD	2	0.14
		ME	3	0.21
		NJ	6	0.43
		NY	3	0.21
		PA	42	2.99
	Plains	KS	3	0.21
		MT	1	0.07
		ND	3	0.21
		SD	1	0.07
	South	FL	7	0.50
		LA	1	0.07
		NC	1	0.07

	NM	1	0.07
	TX	12	0.85
West	UT	1	0.07
Unspecified USA	USA	7	0.50
Outside USA	N/A	925	65.88
Older Sequences		337	24.00

Supplemental Table S4

Class	Genotype	N	Median TMRCA (years)	95% HPD
I	1a	28	16.6	(14.0, 20.2)
	1b	56	11.6	(9.2, 14.7)
	1c	22	23.5	(20.5, 27.3)
	1d	92	28.4	(24.1, 34.3)
	Root Height		50.8	(33.6, 73.6)
II	I	105	49.9	(37.6, 66.2)
	III	2	46.6	(42.5, 55.4)
	IV	4	85.8	(64.9, 115.1)
	V	93	34.2	(31.9, 37.9)
	VI	302	60.0	(52.2, 71.6)
	VII	616	76.1	(62.2, 95.4)
	VIII	3	45.3	(38.4, 54.0)
	IX	6	65.5	(48.6, 94.1)
	X	23	45.7	(36.3, 58.2)
	XI	14	19.0	(13.1, 28.4)
	XII	14	29.8	(19.5, 43.0)
	XIII	50	42.2	(36.6, 49.4)
	XIV	68	34.4	(25.0, 45.6)
	XVI	3	46.9	(31.9, 79.9)
	XVII	82	33.5	(26.3, 42.2)
	XVIII	16	25.8	(18.1, 35.8)
	Root Height		136.3	(112.3, 161.7)

Supplemental Table S5. Class I median diffusion rates between host orders. The 95% Bayesian credibility interval and Bayes factor are provided for each rate.

	Sink		
Source	Anseriformes	Charadriiformes	Chickens
Anseriformes		1.16<br>[0.30, 2.27]<br>11,050.89	0.89<br>[0.00, 4.43]<br>10.86
Charadriiformes	0.74<br>[0.00, 2.23]<br>12.98		0.32<br>[0.00, 1.35]<br>0.87
Chickens	6.18<br>[0.31, 10.02]<br>367.18	0.20<br>[0.00, 0.90]<br>0.55	

Decisive Support | Strong Support | Not Supported

Supplemental Table S6. Class I median diffusion rates between host orders. The 95% Bayesian credibility interval and Bayes factor are provided for each rate.

	Sink					
Source	Anseriformes	Chickens	Columbiformes	Other Galliformes	Psittaciformes	Suliformes
Anseriformes		1.44<br>[0.52, 2.74]<br>34,582.89	0.39<br>[0.00, 1.22]<br>1.44	0.19<br>[0.00, 0.74]<br>0.36	0.26<br>[0.00, 0.82]<br>0.75	0.10<br>[0.00, 0.47]<br>0.18
Chickens	2.88<br>[1.91, 4.06]<br>34,582.89		1.29<br>[0.68, 2.02]<br>34,582.89	1.73<br>[1.06, 2.60]<br>34,582.89	0.57<br>[0.21, 1.04]<br>34,582.89	0.06<br>[0.00, 0.26]<br>0.06
Columbiformes	0.49<br>[0.07, 1.13]<br>322.02	0.44<br>[0.05, 1.03]<br>402.64		0.24<br>[0.00, 0.68]<br>3.55	0.12<br>[0.00, 0.49]<br>0.23	0.08<br>[0.00, 0.36]<br>0.14
Other Galliformes	0.33<br>[0.00, 1.15]<br>0.99	0.60<br>[0.00, 1.80]<br>1.42	0.23<br>[0.00, 1.00]<br>0.44		0.43<br>[0.01, 1.17]<br>9.57	0.14<br>[0.00, 0.57]<br>0.23
Psittaciformes	0.39<br>[0.00, 1.14]<br>4.31	0.39<br>[0.00, 1.27]<br>2.57	0.24<br>[0.00, 0.97]<br>0.43	0.32<br>[0.00, 0.98]<br>1.37		0.12<br>[0.00, 0.52]<br>0.21
Suliformes	0.14<br>[0.00, 0.59]<br>0.25	0.18<br>[0.00, 0.67]<br>0.31	0.14<br>[0.00, 0.59]<br>0.26	0.16<br>[0.00, 0.63]<br>0.18	0.16<br>[0.00, 0.59]<br>0.21	

Decisive Support   Substantial Support   Not Supported

Supplemental Table S7. Class I median diffusion rates between world geographic regions. The 95% Bayesian credibility interval and Bayes factor are provided for each rate.

	Sink			
Source	CentralAsia	EastAsia	Europe	NorthAmerica
CentralAsia		0.40<br>[0.00, 1.92]<br>0.46	0.38<br>[0.00, 1.60]<br>0.40	0.41<br>[0.00, 1.82]<br>0.43
EastAsia	0.44<br>[0.00, 1.39]<br>1.75		0.47<br>[0.01, 1.37]<br>18.91	0.21<br>[0.00, 0.91]<br>0.21
Europe	0.41<br>[0.00, 1.81]<br>0.45	0.44<br>[0.00, 1.95]<br>0.50		0.40<br>[0.00, 1.83]<br>0.42
NorthAmerica	0.21<br>[0.00, 1.05]<br>0.24	0.23<br>[0.00, 1.14]<br>0.23	0.22<br>[0.00, 1.05]<br>0.23	

Strong Support   Not Supported

Supplemental Table S8. Class II median diffusion rates between world geographic regions. The 95% Bayesian credibility interval and Bayes factor are provided for each rate.

	Sink									
Source	Africa	CentralAmerica	CentralAsia	EastAsia	Europe	MiddleEast	NorthAmerica	SouthAmerica	SouthAsia	SoutheastAsia
Africa		0.16<br>[0.01, 0.62]<br>0.16	0.21<br>[0.01, 0.83]<br>0.34	0.29<br>[0.00, 0.94]<br>5.40	0.23<br>[0.00, 1.07]<br>0.31	0.15<br>[0.00, 0.63]<br>0.17	0.13<br>[0.01, 0.68]<br>0.23	0.09<br>[0.00, 0.54]<br>0.19	0.19<br>[0.01, 0.66]<br>0.16	0.13<br>[0.00, 0.46]<br>0.21
CentralAmerica	0.28<br>[0.01, 1.09]<br>0.32		0.27<br>[0.01, 0.91]<br>0.29	0.27<br>[0.01, 1.14]<br>0.40	0.27<br>[0.00, 1.00]<br>0.27	0.13<br>[0.00, 0.78]<br>0.40	0.20<br>[0.00, 0.82]<br>0.35	0.26<br>[0.00, 1.10]<br>0.33	0.26<br>[0.01, 1.22]<br>0.34	0.22<br>[0.00, 0.85]<br>0.31
CentralAsia	0.60<br>[0.01, 2.01]<br>1.07	0.25<br>[0.01, 0.95]<br>0.37		0.69<br>[0.04, 1.91]<br>96.26	0.71<br>[0.01, 2.03]<br>37.03	0.26<br>[0.00, 1.15]<br>0.31	0.18<br>[0.00, 0.91]<br>0.39	0.21<br>[0.02, 0.92]<br>0.33	0.31<br>[0.00, 1.23]<br>0.31	0.31<br>[0.01, 1.13]<br>0.30
EastAsia	0.29<br>[0.00, 0.85]<br>1.08	0.13<br>[0.01, 0.59]<br>0.13	0.10<br>[0.00, 0.43]<br>0.21		0.19<br>[0.01, 0.99]<br>0.29	0.66<br>[0.14, 1.35]<br>8,381.22	0.23<br>[0.01, 0.69]<br>0.25	0.14<br>[0.01, 0.73]<br>0.15	0.11<br>[0.01, 0.58]<br>0.15	0.09<br>[0.00, 0.42]<br>0.23
Europe	1.06<br>[0.07, 2.51]<br>169.27	0.23<br>[0.01, 0.95]<br>0.25	0.66<br>[0.00, 1.97]<br>11.78	1.17<br>[0.12, 2.61]<br>436.19		0.37<br>[0.01, 1.33]<br>0.49	0.16<br>[0.00, 0.85]<br>0.32	0.18<br>[0.00, 0.77]<br>0.34	0.50<br>[0.00, 1.63]<br>1.24	0.14<br>[0.00, 0.78]<br>0.30
MiddleEast	0.30<br>[0.00, 1.05]<br>0.34	0.14<br>[0.00, 0.87]<br>0.35	0.20<br>[0.00, 0.81]<br>0.29	0.42<br>[0.00, 1.62]<br>1.02	0.25<br>[0.00, 1.00]<br>0.34		0.25<br>[0.02, 0.93]<br>0.32	0.19<br>[0.00, 0.86]<br>0.28	0.26<br>[0.00, 1.22]<br>0.42	0.29<br>[0.01, 1.24]<br>0.34
NorthAmerica	0.22<br>[0.01, 0.68]<br>0.24	0.14<br>[0.00, 0.90]<br>0.22	0.19<br>[0.00, 0.68]<br>0.25	0.45<br>[0.01, 1.52]<br>0.61	0.23<br>[0.01, 0.80]<br>0.25	0.19<br>[0.01, 0.71]<br>0.26		0.16<br>[0.00, 0.78]<br>0.29	0.21<br>[0.00, 0.87]<br>0.19	0.12<br>[0.00, 0.62]<br>0.35
SouthAmerica	0.23<br>[0.00, 0.95]<br>0.40	0.20<br>[0.00, 0.89]<br>0.38	0.22<br>[0.00, 1.09]<br>0.33	0.31<br>[0.01, 1.16]<br>0.39	0.30<br>[0.00, 1.14]<br>0.29	0.24<br>[0.01, 0.94]<br>0.39	0.23<br>[0.00, 1.03]<br>0.39		0.26<br>[0.00, 1.02]<br>0.36	0.24<br>[0.01, 0.85]<br>0.33
SouthAsia	0.25<br>[0.00, 1.17]<br>0.64	0.16<br>[0.01, 0.85]<br>0.23	0.15<br>[0.00, 0.80]<br>0.26	0.24<br>[0.01, 0.94]<br>0.24	0.47<br>[0.02, 1.35]<br>13.10	1.21<br>[0.36, 2.45]<br>67,107.77	0.18<br>[0.01, 0.90]<br>0.22	0.17<br>[0.00, 0.70]<br>0.28		0.45<br>[0.01, 1.40]<br>47.18
SoutheastAsia	0.55<br>[0.00, 1.64]<br>8.72	0.22<br>[0.00, 0.87]<br>0.36	0.20<br>[0.00, 1.05]<br>0.33	0.51<br>[0.01, 1.84]<br>1.47	0.29<br>[0.00, 1.17]<br>0.48	0.31<br>[0.00, 1.05]<br>0.49	0.25<br>[0.01, 1.07]<br>0.32	0.28<br>[0.00, 1.07]<br>0.29	0.55<br>[0.01, 1.65]<br>31.67	

Decisive Support · Very Strong Support · Strong Support · Substantial Support · Not Supported

Supplemental Table S9. Class I median migration rates into, out of and within the United States. The 95% Bayesian credibility interval and Bayes factor are provided for each rate.

	Sink					
Source	Alaska	Midwest	Northeast	South	West	Outside United States
Alaska		3.24<br>[0.95, 6.13]<br>8,642.5	2.63<br>[0.58, 5.25]<br>2,466.2	1.28<br>[0.01, 3.17]<br>12.7	0.34<br>[0.00, 1.45]<br>0.5	0.28<br>[0.00, 1.34]<br>0.5
Midwest	0.68<br>[0.00, 2.28]<br>1.6		1.94<br>[0.19, 4.45]<br>402.6	1.20<br>[0.00, 3.10]<br>8.3	0.36<br>[0.00, 1.50]<br>0.6	0.33<br>[0.00, 1.52]<br>0.5
Northeast	0.40<br>[0.00, 1.79]<br>0.7	1.55<br>[0.06, 3.98]<br>91.8		1.46<br>[0.09, 3.72]<br>149.5	0.53<br>[0.00, 1.95]<br>0.9	0.38<br>[0.00, 1.68]<br>0.5
South	0.39<br>[0.00, 1.67]<br>0.6	0.51<br>[0.00, 2.26]<br>0.8	0.47<br>[0.00, 2.00]<br>0.8		0.91<br>[0.00, 2.59]<br>23.7	0.42<br>[0.00, 1.86]<br>0.6
West	0.42<br>[0.00, 1.96]<br>0.6	0.43<br>[0.00, 1.92]<br>0.7	0.40<br>[0.00, 1.68]<br>0.6	0.56<br>[0.00, 2.19]<br>0.8		0.43<br>[0.00, 1.83]<br>0.6
Outside United States	0.15<br>[0.00, 0.72]<br>0.2	0.15<br>[0.00, 0.85]<br>0.3	0.16<br>[0.00, 0.83]<br>0.3	0.16<br>[0.00, 0.68]<br>0.2	0.15<br>[0.00, 0.67]<br>0.2	

Decisive Support | Strong Support | Not Supported

Very Strong Support | Substantial Support

Supplemental Table S10. Class I median migration rates into, out of and within the United States. The 95% Bayesian credibility interval and Bayes factor are provided for each rate.

	Sink						
Source	Alaska	Midwest	Northeast	Plains	South	West	Outside United States
Alaska		0.36<br>[0.00, 1.15]<br>0.67	0.40<br>[0.01, 1.20]<br>4.32	0.15<br>[0.00, 0.67]<br>0.18	0.18<br>[0.00, 0.82]<br>0.31	0.17<br>[0.00, 0.68]<br>0.22	0.25<br>[0.00, 0.92]<br>0.29
Midwest	0.28<br>[0.00, 0.90]<br>0.51		0.29<br>[0.00, 1.28]<br>0.40	0.28<br>[0.00, 0.98]<br>0.65	1.35<br>[0.28, 2.69]<br>62.34	0.19<br>[0.00, 0.67]<br>0.16	0.22<br>[0.00, 1.00]<br>0.26
Northeast	0.15<br>[0.00, 0.67]<br>0.22	1.91<br>[0.77, 3.34]<br>42,728.11		0.38<br>[0.00, 1.19]<br>2.09	0.52<br>[0.02, 1.41]<br>11.65	0.10<br>[0.00, 0.57]<br>0.19	0.23<br>[0.00, 0.84]<br>0.22
Plains	0.17<br>[0.00, 0.65]<br>0.19	0.32<br>[0.00, 1.09]<br>0.97	0.29<br>[0.01, 1.14]<br>0.89		0.18<br>[0.00, 0.75]<br>0.20	0.21<br>[0.00, 0.87]<br>0.30	0.20<br>[0.00, 1.07]<br>0.26
South	0.16<br>[0.00, 0.85]<br>0.22	0.68<br>[0.00, 2.11]<br>2.70	0.35<br>[0.00, 1.13]<br>0.67	0.85<br>[0.17, 1.84]<br>8,541.40		0.33<br>[0.00, 1.03]<br>6.42	0.19<br>[0.00, 0.86]<br>0.29
West	0.14<br>[0.00, 0.71]<br>0.21	0.17<br>[0.00, 0.68]<br>0.21	0.14<br>[0.00, 0.66]<br>0.23	0.20<br>[0.00, 0.73]<br>0.21	0.16<br>[0.00, 0.68]<br>0.22		0.26<br>[0.01, 1.04]<br>0.29
Outside United States	0.03<br>[0.00, 0.18]<br>0.06	0.06<br>[0.00, 0.20]<br>0.04	0.05<br>[0.01, 0.17]<br>0.05	0.05<br>[0.00, 0.17]<br>0.06	0.05<br>[0.00, 0.27]<br>0.04	0.04<br>[0.00, 0.20]<br>0.04	

Decisive Support | Very Strong Support | Strong Support | Substantial Support | Not Supported

Supplemental Table S11

Class		Trait	Main Model	Tip Swap
I	World	Europe	0.01	0.00
		Central Asia	0.26	0.00
		East Asia	**0.72**	0.39
		North America	0.01	**0.54**
		Older Sequence	0.01	0.06
	Host	Chickens	0.28	0.07
		Anseriformes	0.15	**0.75**
		Charadriiformes	0.06	0.03
		Older Sequence	**0.52**	0.16
	US Region	South	0.01	0.09
		Northeast	0.01	0.15
		West	0.01	0.02
		Non-US	**0.79**	0.26
		Alaska	0.01	0.06
		Midwest	0.01	0.12
		Older Sequence	0.18	**0.30**
II	World	South Asia	0.01	0.02
		Europe	0.02	0.02
		Central America	0.07	0.02
		Africa	0.02	0.05
		South America	0.01	0.00
		Southeast Asia	0.02	0.00
		Middle East	0.01	0.00
		East Asia	0.01	0.18
		North America	0.01	0.05
		Central Asia	0.01	0.00
		Older	**0.81**	**0.66**
	Host	Columbiformes	0.02	0.10
		Galliformes	0.02	0.00
		Chickens	0.03	0.22
		Anseriformes	0.03	0.04
		Psittaciformes	0.02	0.02
		Suliformes	0.02	0.00
		**Older Sequence**	**0.84**	**0.64**
	US Region	Plains	0.02	0.00
		South	0.02	0.00
		Northeast	0.02	0.01
		West	0.02	0.00

Non-US	0.09	0.49
Midwest	0.02	0.00
Alaska	0.02	0.00
Older Sequence	**0.80**	**0.51**
