# Supplementary material for: Global phylodynamic analysis of avian paramyxovirus-1 provides evidence of inter-host transmission and intercontinental spatial diffusion
Source: BMC Evol Biol. 2019 May 24;19:108. doi: 10.1186/s12862-019-1431-2 (PMC6534909; doi:10.1186/s12862-019-1431-2)

TITLE: Global phylodynamic analysis of avian paramyxovirus-1 provides evidence of inter-host transmission and intercontinental spatial diffusion

AUTHORS: Joseph T. Hicks[1], Kiril M. Dimitrov[2], Claudio L. Afonso[2], Andrew M. Ramey[3], and Justin Bahl[1,4]

[1]University of Georgia College of Veterinary Medicine Department of Infectious Diseases, Athens, Georgia, USA

[2]Exotic and Emerging Avian Viral Disease Research Unit, Southeast Poultry Research Laboratory, US National Poultry Research Center, ARS, USDA, Athens, GA, USA

[3]US Geological Survey, Alaska Science Center, Anchorage, AK, USA

[4]Program in Emerging Infectious Diseases, Duke-National University of Singapore Graduate Medical School, 8 College Road, Singapore 169857, Singapore

For Correspondence Contact: Joseph Hicks: Joseph.Hicks@uga.edu or Justin Bahl: Justin.Bahl@uga.edu

Address: 501 D. W. Brooks Drive, Athens, Georgia, 30602

Please find below descriptions and figures for Additional File 2.

Supplemental Figures

Supplemental Figure S1. Global Distribution of Newcastle Disease (ND), 2005 - 2016 (A). Countries with at least one ND outbreak are indicated by blue circles. Blue crosses indicate suspected, but unconfirmed Newcastle Disease cases. APMV-1 complete fusion gene sequences collected between 2006 and 2016 and included in this analysis originated from the shaded countries. Base map was used with permission from OpenStreetMap (https://www.openstreetmap.org/copyright). For comparison, the distribution of common hosts, domestic chickens (B) and domestic ducks (C), are provided from the Gridded Livestock of the World database (GLW v3.1) of the Food and Agriculture Organization of the United Nations (FAO) (B: Gilbert, Marius; Nicolas, Gaëlle; Cinardi, Giusepina; Van Boeckel, Thomas P.; Vanwambeke, Sophie; Wint, G. R. William; Robinson, Timothy P., 2018, "Global chickens distribution in 2010 (5 minutes of arc)", https://doi.org/10.7910/DVN/SUFASB, Harvard Dataverse, V3; C: Gilbert, Marius; Nicolas, Gaëlle; Cinardi, Giusepina; Van Boeckel, Thomas P.; Vanwambeke, Sophie; Wint, William G. R.; Robinson, Timothy P., 2018, "Global ducks distribution in 2010 (5 minutes of arc)", https://doi.org/10.7910/DVN/ICHCBH, Harvard Dataverse, V3). (**Page 4**)

Supplemental Figure S2. Class I Host Transition Heat Map, 2005 - 2016. Darkness of each cell color is proportional to the absolute number of transitions from the first column into the second column. The maximum transition count is 6.0 transitions (Domestic chickens to Anseriformes, 2009). (ANS – Anseriformes, CHA – Charadriformes, CHI – domestic chicken) (**Page 5**)

Supplemental Figure S3. Class II Host Transition Heat Map, 2005-2016. Darkness of each cell color is proportional to the absolute number of transitions from the first column into the second column. The maximum transition count is 9.6 transitions (Domestic chickens to Anseriformes, 2009). (ANS – Anseriformes, CHI – domestic chicken, COL – Columbiformes, GAL – other Galliformes, PSI - Psittaciformes) (**Page 6**)

Supplemental Figure S4. Class I World Migration Heat Map, 2005-2016. Darkness of each cell color is proportional to the absolute number of transitions from the first column into the second column. The maximum migration count is 0.92 migrations (East Asia to Europe, 2006). (**Page 7**)

Supplemental Figure S5. Class II World Migration Heat Map, 2005-2016. Darkness of each cell color is proportional to the absolute number of transitions from the first column into the second column. The maximum migration count is 1.3 migrations (South Asia to Middle East, 2011). (**Page 8**)

Supplemental Figure S6. Tip Swap Analysis Host Transition Rates. Blue circles indicate the median transition rate per year from the host source (top axis label) to the sink (bottom axis label). Gray bars represent the 95% highest posterior density of the estimate. The “Older” category represents sequences collected before 2006 of any host. (ANS – Anseriformes, CHA – Charadriformes, CHI – domestic chicken, COL – Columbiformes, GAL – other Galliformes, PSI - Psittaciformes) (**Page 9**)

Supplemental Figure S7. Tip Swap Analysis World Region Transition Rates. Blue circles indicate the median transition rate per year from the world region source (top axis label) to the sink (bottom axis label). Gray bars represent the 95% highest posterior density of the estimate. The “Older/UN” category represents sequences collected before 2006 of any host. (**Page 10**)

Supplemental Figure S8. Tip Swap Analysis United States Transition Rates. Blue circles indicate the median transition rate per year from the United States region source (top axis label) to the sink (bottom axis label). Gray bars represent the 95% highest posterior density of the estimate. The “Older/UN” category represents sequences collected before 2006 of any host. (AK – Alaska, MW – Midwest, NE – Northeast, P – Plains, S – South, W- West, X – Outside United States) (**Page 11**)

Supplemental Figure S1. Global Distribution of Newcastle Disease (ND), 2005 - 2016 (A). Countries with at least on ND outbreak are indicated by blue circles. Blue crosses indicate suspected, but unconfirmed Newcastle Disease cases. APMV-1 complete fusion gene sequences collected between 2006 and 2016 and included in this analysis originated from the shaded countries. Base map was used with permission from OpenStreetMap (https://www.openstreetmap.org/copyright). For comparison, the distribution of common hosts, domestic chickens (B) and domestic ducks (C), are provided from the Gridded Livestock of the World database (GLW v3.1) of the Food and Agriculture Organization of the United Nations (FAO) (B: Gilbert, Marius; Nicolas, Gaëlle; Cinardi, Giusepina; Van Boeckel, Thomas P.; Vanwambeke, Sophie; Wint, G. R. William; Robinson, Timothy P., 2018, "Global chickens distribution in 2010 (5 minutes of arc)", https://doi.org/10.7910/DVN/SUFASB, Harvard Dataverse, V3; C: Gilbert, Marius; Nicolas, Gaëlle; Cinardi, Giusepina; Van Boeckel, Thomas P.; Vanwambeke, Sophie; Wint, William G. R.; Robinson, Timothy P., 2018, "Global ducks distribution in 2010 (5 minutes of arc)", https://doi.org/10.7910/DVN/ICHCBH, Harvard Dataverse, V3).

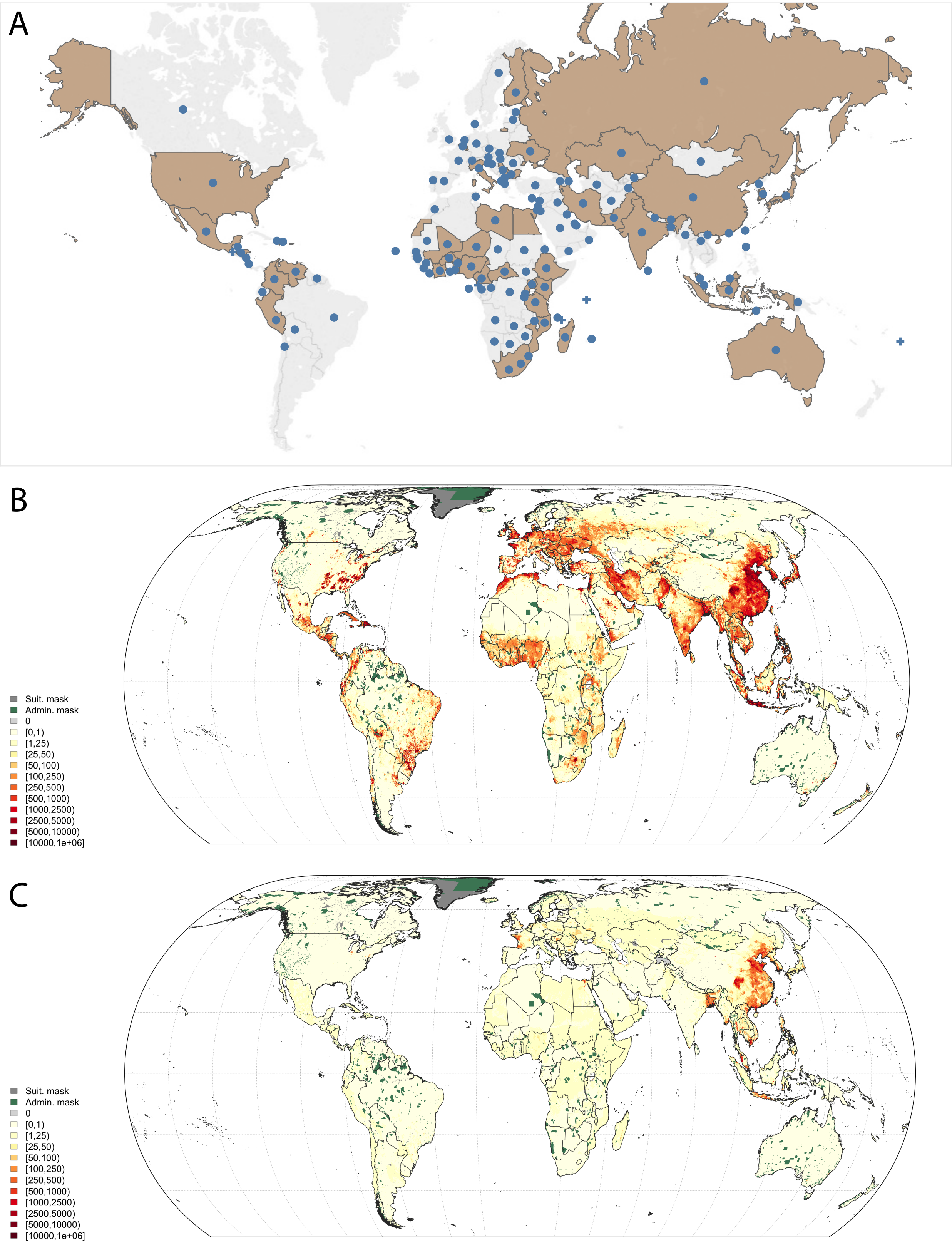

Supplemental Figure S2. Class I Host Transition Heat Map, 2005-2016. Darkness of each cell color is proportional to the absolute number of transitions from the first column into the second column. The maximum transition count is 6.0 transitions (Domestic chickens to Anseriformes, 2009). (ANS – Anseriformes, CHA – Charadriiformes, CHI – domestic chicken)

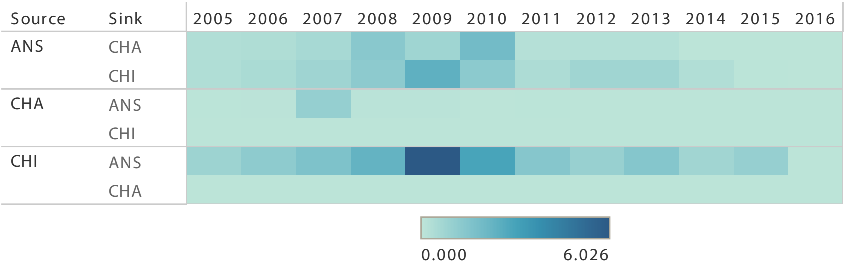

Supplemental Figure S3. Class II Host Transition Heat Map, 2005-2016. Darkness of each cell color is proportional to the absolute number of transitions from the first column into the second column. The maximum transition count is 9.6 transitions (Domestic chickens to Anseriformes, 2009). (ANS – Anseriformes, CHI – domestic chicken, COL – Columbiformes, GAL – other Galliformes, PSI - Psittaciformes)

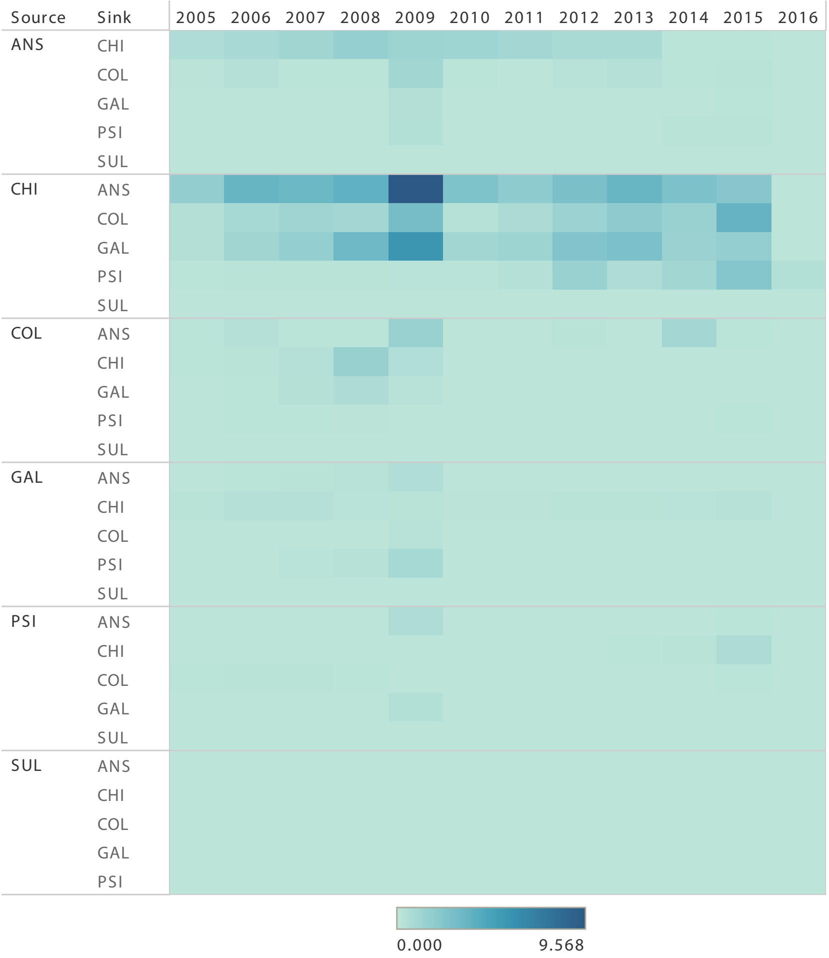

Supplemental Figure S4. Class I World Migration Heat Map, 2005-2016. Darkness of each cell color is proportional to the absolute number of transitions from the first column into the second column. The maximum migration count is 0.92 migrations (East Asia to Europe, 2006).

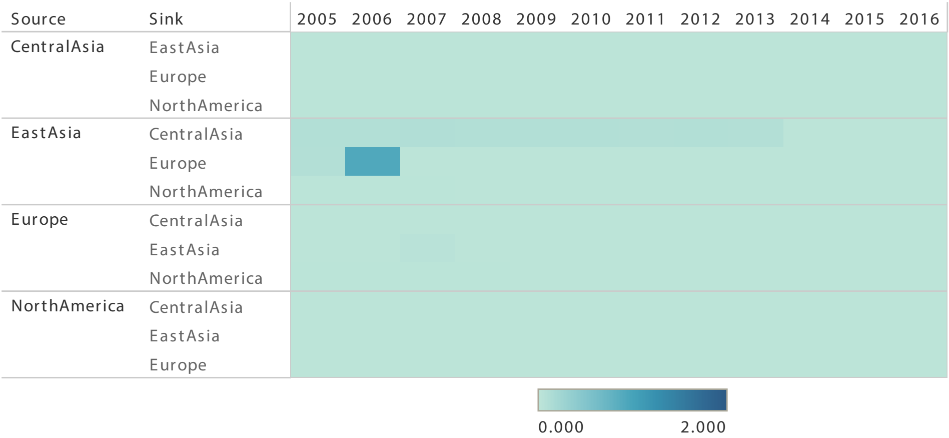

Supplemental Figure S5. Class II World Migration Heat Map, 2005-2016. Darkness of each cell color is proportional to the absolute number of transitions from the first column into the second column. The maximum migration count is 1.3 migrations (South Asia to Middle East, 2011).

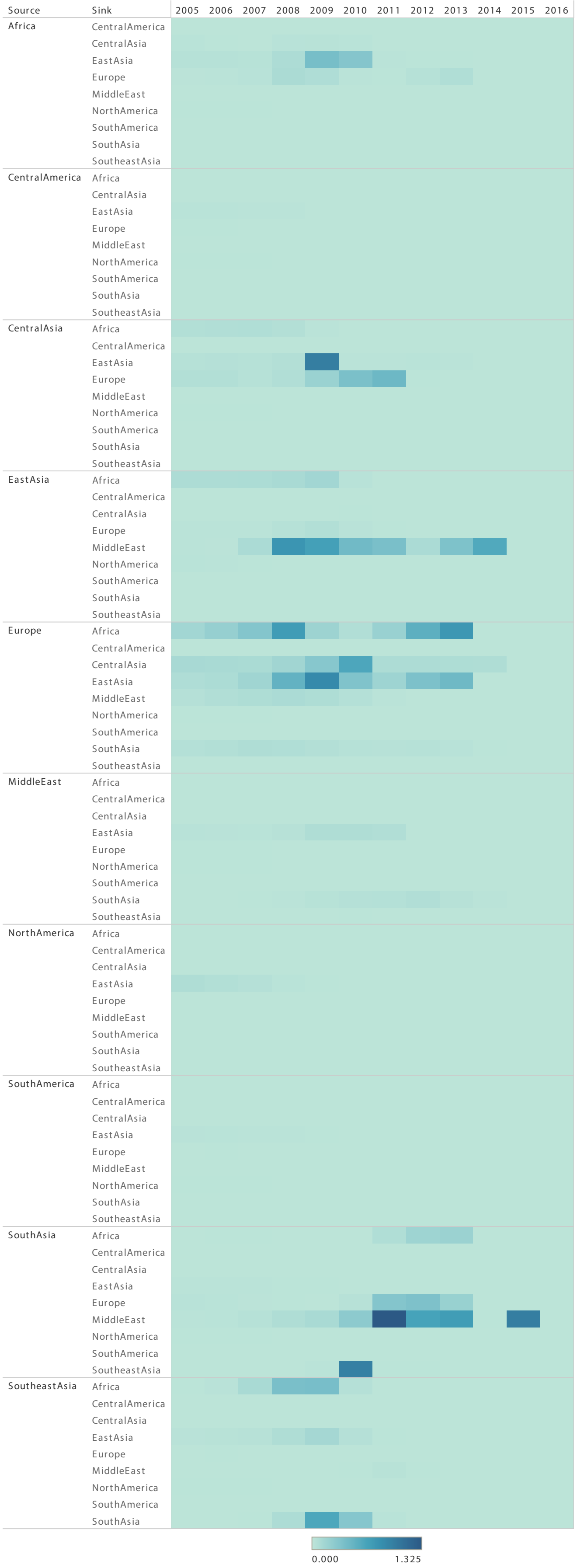

Supplemental Figure S6. Tip Swap Analysis Host Transition Rates. Blue circles indicate the median transition rate per year from the host source (top axis label) to the sink (bottom axis label). Gray bars represent the 95% highest posterior density of the estimate. The "Older" category represents sequences collected before 2006 of any host. (ANS – Anseriformes, CHA – Charadriformes, CHI – domestic chicken, COL – Columbiformes, GAL – other Galliformes, PSI - Psittaciformes)

Class I

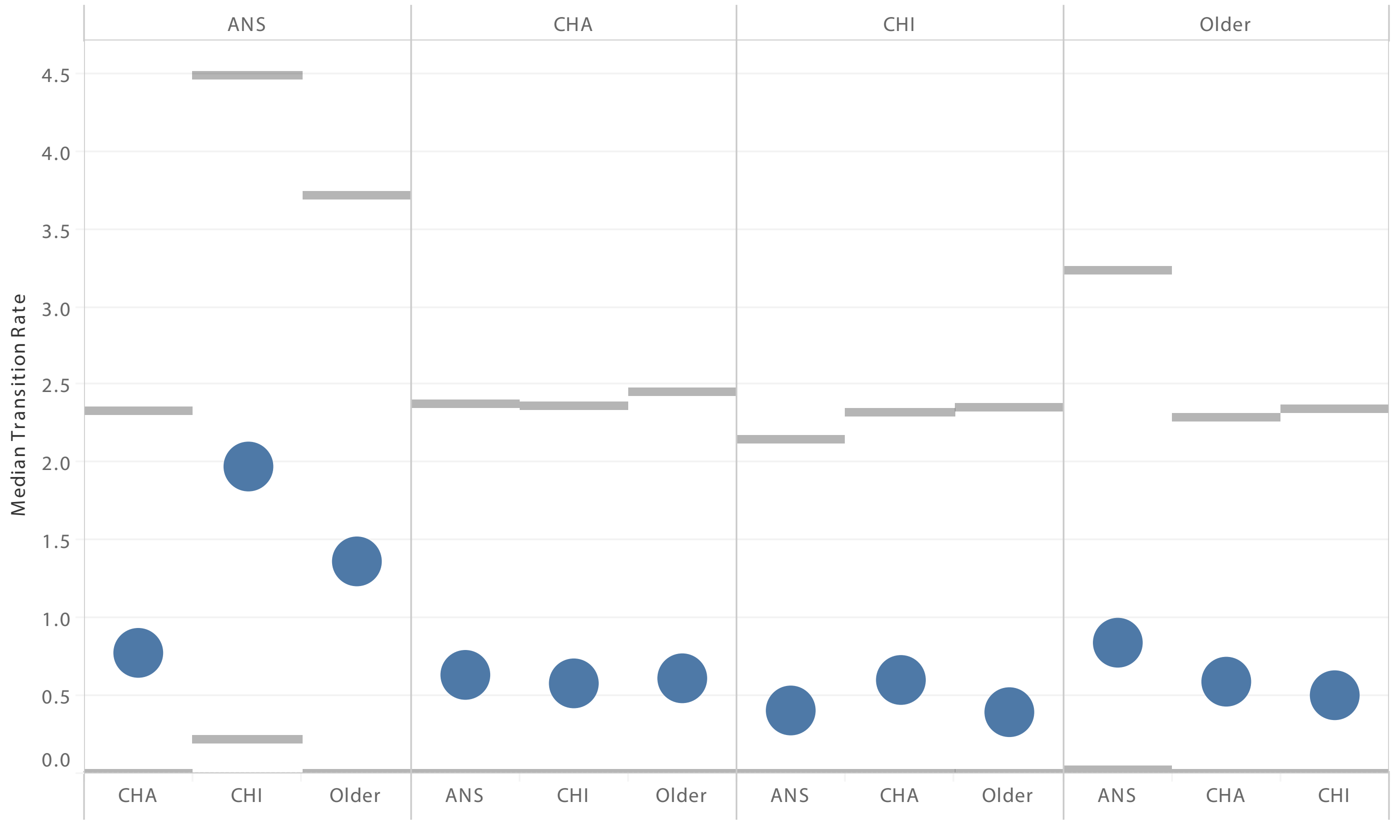

Class II

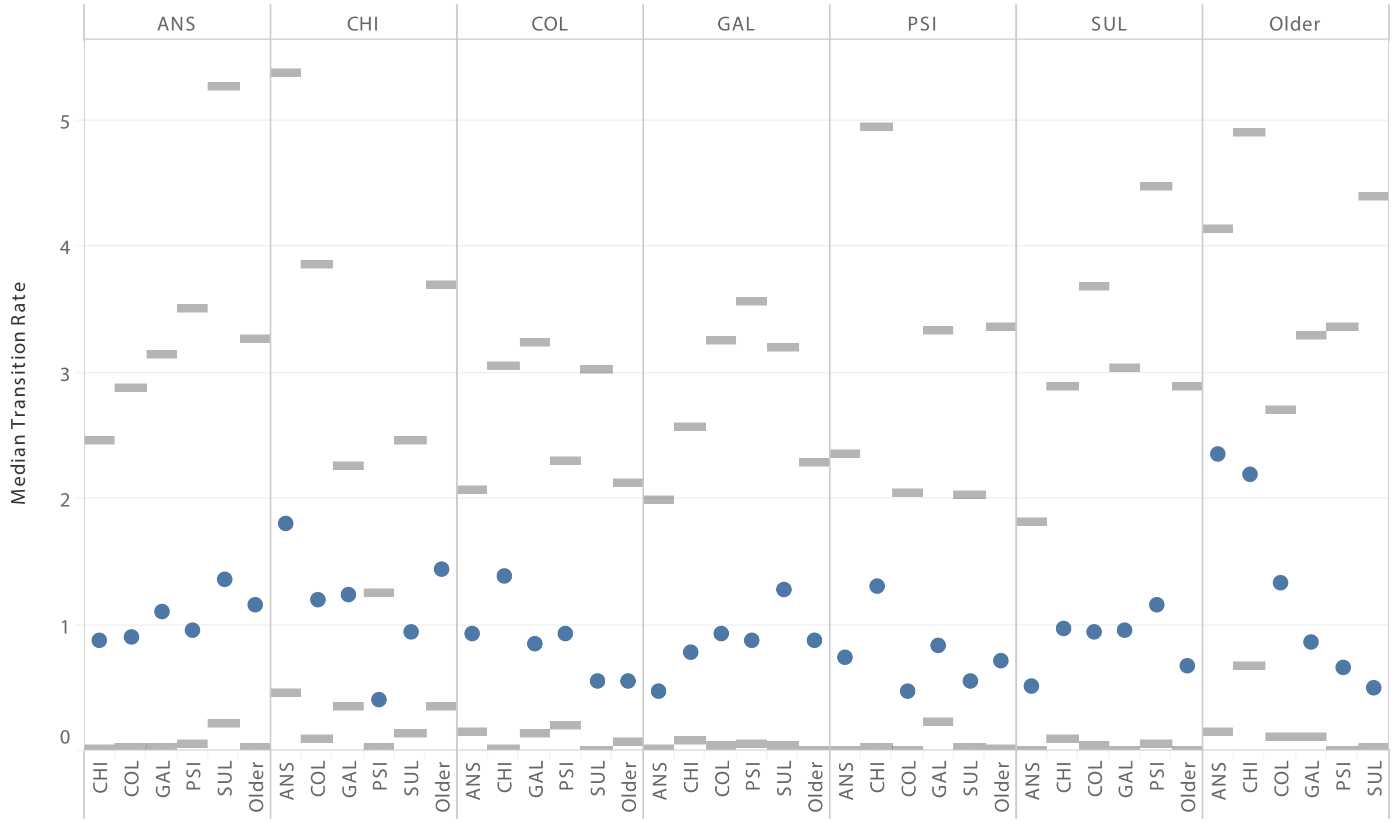

Supplemental Figure S7. Tip Swap Analysis World Region Transition Rates. Blue circles indicate the median transition rate per year from the world region source (top axis label) to the sink (bottom axis label). Gray bars represent the 95% highest posterior density of the estimate. The "Older/UN" category represents sequences collected before 2006 of any host.

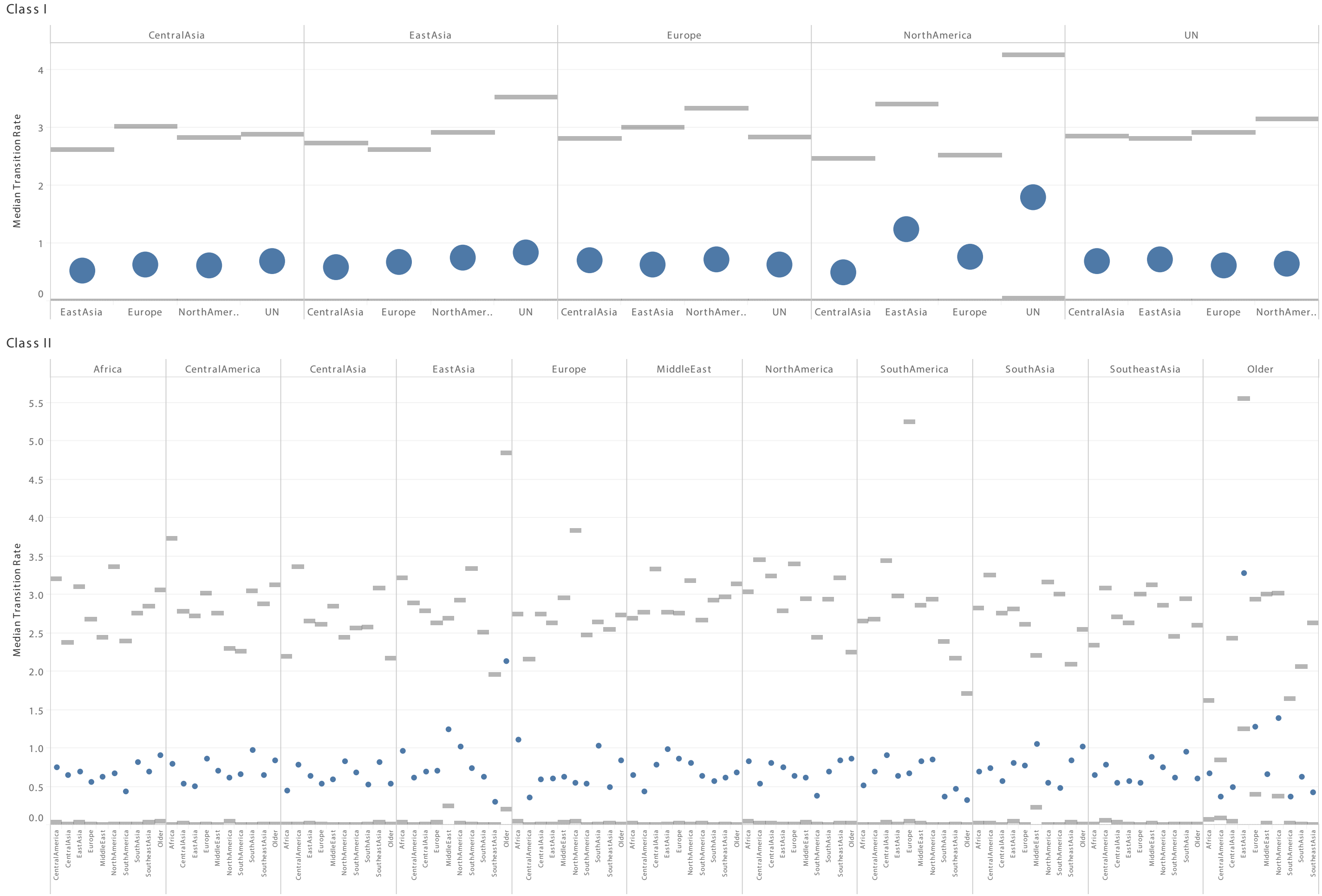

Supplemental Figure S8. Tip Swap Analysis United States Transition Rates. Blue circles indicate the median transition rate per year from the United States region source (top axis label) to the sink (bottom axis label). Gray bars represent the 95% highest posterior density of the estimate. The "Older/UN" category represents sequences collected before 2006 of any host. (AK – Alaska, MW – Midwest, NE – Northeast, P – Plains, S – South, W- West, X – Outside United States)

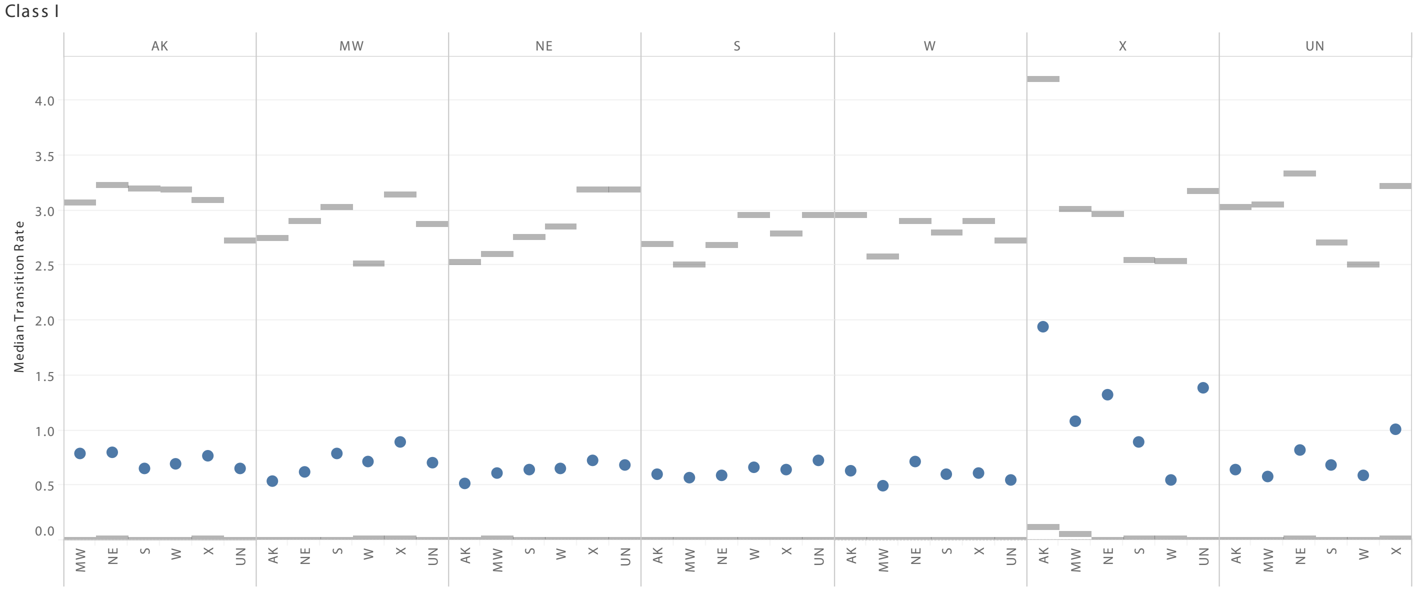

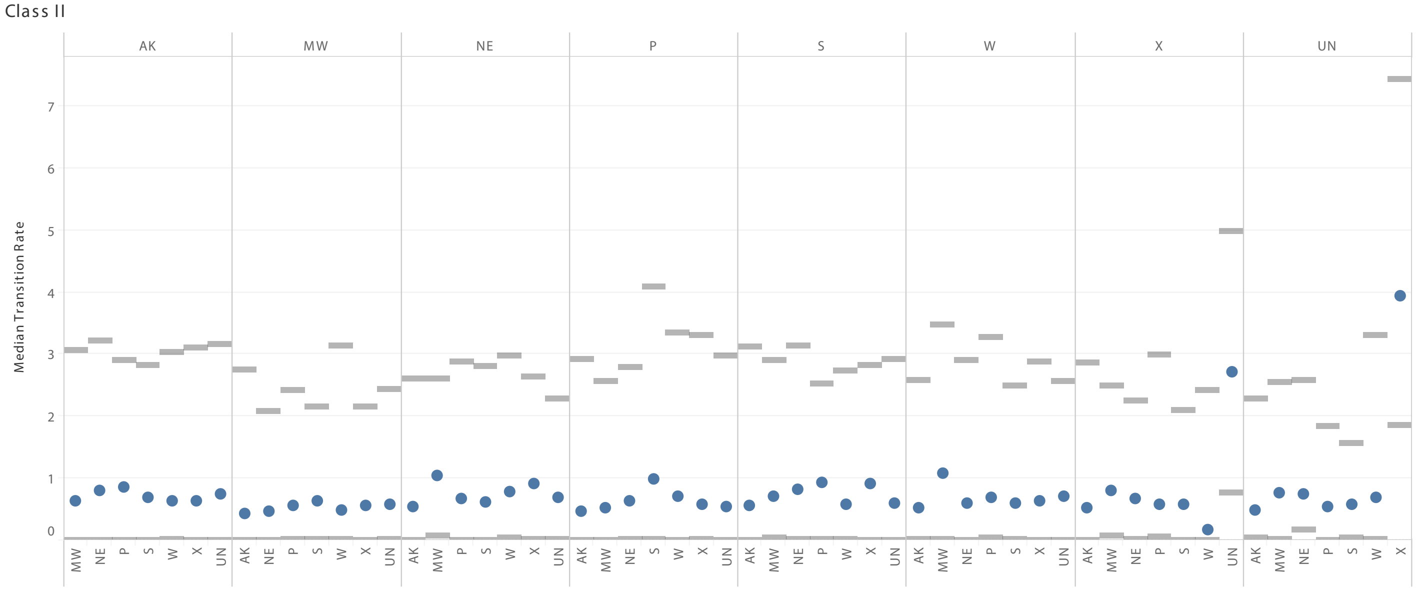

Supplement: Supplementary file 2 — Figures S1 - S8. (PDF 8119 kb) [file 12862_2019_1431_MOESM2_ESM.pdf]
